# Coumarin as an Elite Scaffold in Anti-Breast Cancer Drug Development: Design Strategies, Mechanistic Insights, and Structure–Activity Relationships

**DOI:** 10.3390/biomedicines12061192

**Published:** 2024-05-27

**Authors:** Atamjit Singh, Karanvir Singh, Kamaljit Kaur, Amandeep Singh, Aman Sharma, Kirandeep Kaur, Jaskirat Kaur, Gurleen Kaur, Uttam Kaur, Harsimran Kaur, Prabhsimran Singh, Preet Mohinder Singh Bedi

**Affiliations:** 1 Department of Pharmaceutical Sciences, Guru Nanak Dev University, Amritsar 143005, Punjab, India; karanvirsinghrajput@gmail.com (K.S.); aman.pharma.rsh@gmail.com (A.S.); kaurkirandeep219@gmail.com (K.K.); ghumanjaskiratkaur@gmail.com (J.K.); gurleenkaur07385@gmail.com (G.K.); 2 Hershey Dental Group, Hershey, PA 17033, USA; drkamal1988@gmail.com; 3 Department of Pharmacology, Penn State Cancer Institute, CH72, Penn State College of Medicine, Penn State Milton S. Hershey Medical Center, 500 University Drive, Hershey, PA 17033, USA; asingh8@pennstatehealth.psu.edu; 4 University School of Business Management, Chandigarh University, Gharuan 140413, Mohali, India; uttamkaur7@gmail.com; 5 Department of Pharmaceutical Chemistry, Khalsa College of Pharmacy, Amritsar 143005, Punjab, India; harsimranksabharwal@gmail.com (H.K.); prabh.7750@gmail.com (P.S.); 6 Drug and Pollution Testing Laboratory, Guru Nanak Dev University, Amritsar 143005, Punjab, India

**Keywords:** coumarin, anticancer, breast cancer, drug development, structure–activity relationship

## Abstract

Breast cancer is the most common cancer among women. Currently, it poses a significant threat to the healthcare system due to the emerging resistance and toxicity of available drug candidates in clinical practice, thus generating an urgent need for the development of new potent and safer anti-breast cancer drug candidates. Coumarin (chromone-2-one) is an elite ring system widely distributed among natural products and possesses a broad range of pharmacological properties. The unique distribution and pharmacological efficacy of coumarins attract natural product hunters, resulting in the identification of numerous natural coumarins from different natural sources in the last three decades, especially those with anti-breast cancer properties. Inspired by this, numerous synthetic derivatives based on coumarins have been developed by medicinal chemists all around the globe, showing promising anti-breast cancer efficacy. This review is primarily focused on the development of coumarin-inspired anti-breast cancer agents in the last three decades, especially highlighting design strategies, mechanistic insights, and their structure–activity relationship. Natural coumarins having anti-breast cancer efficacy are also briefly highlighted. This review will act as a guideline for researchers and medicinal chemists in designing optimum coumarin-based potent and safer anti-breast cancer agents.

## 1. Introduction

Novel therapeutic interventions, as well as preventive strategies, along with a deep understanding of the disease pathogenesis over the last five decades, have resulted in a significant decrease in the primary disease conditions that humans encounter frequently, including cardiovascular diseases, stroke, pneumonia, etc. [1,2]. At the same time, the death curve in cancer has not changed much. Cancer remains the most prevalent and the primary issue for healthcare setups across the globe [3]. Among the cancer cases, breast cancer is among the foremost contributors and is the fifth most prevalent cause of cancer deaths. Breast cancer primarily affects women, with a small percentage of men (0.5–1%) [4]. As per WHO, in 2022, approximately 2.3 million women were diagnosed with breast cancer, which is responsible for 670,000 deaths around the globe. It can thus be considered a global emergency that needs to be tackled immediately [5,6]. 

Breast cancer usually occurs after puberty in women and its prevenance is observed to be increased in later life. Approximately half of the breast cancer cases occur in women aged 40 and above, who are highly susceptible to the risk of breast cancer. Numerous risk factors leading to the development of breast cancer include smoking, aging, obesity, radiation exposure, alcohol, postmenopausal hormonal therapy, and reproductive history (including the age of the first menstrual period and the age of the first pregnancy) [7]. Breast cancer risk is potentially increased by inherited “high penetrance” gene mutations, including PALB-2, BRCA1, and BRCA2 [8]. The survival rate among high-income countries is significantly higher (90%) as compared to middle and low-income countries such as India (66%) and South Africa (40%) [9]. 

Heterogenicity among breast cancers is a primary cause that makes their diagnosis and management somewhat difficult [10]. Based on the currently available molecular biomarkers that include the positivity of hormone receptors, i.e., the oestrogen receptor (ER) and the progesterone receptor (PR), and the status of human epidermal growth factor receptor 2 (HER2), as well as proliferation index of Ki67, breast cancer can be classified into four major types, i.e., luminal A, luminal B, basal-like, and overexpressed HER2 tumours [11]. Luminal A is described as ER positive, PR, and HER2 negative, with less than 14% Ki67 presence. Luminal B is described as ER-positive and/or PR-positive, with more than 14% Ki67 presence, and can be further divided into two subclasses based on the positivity and negativity of the HER2 groups. Basal-like tumours have triple-negative receptors, with varying percentages of Ki67. Finally, the fourth category consists of tumours with overexpressed HER2, as well as ER and PR negativity [12,13]. Such diversity in biomarkers also poses significant difficulty in medicine selection and dosage regimen determination, and errors usually lead to poor patient outcomes and drug resistance [10].

Currently, the available drugs for the management of breast cancer either induce unbearable side effects or present toxicity-like issues, along with emerging drug resistance to all available drug candidates. This makes the treatment phase very difficult for both healthcare professionals and patients [14]. On the other hand, natural products offer a wide range of pharmacophores that can be efficiently modified as needed to get the desired level of pharmacological output, along with considerable safety. Coumarin is one such pharmacophore [15,16]. Coumarin (2*H*-1-benzopyran-2-one) was first isolated from the tonka bean (*Dipteryx odorata*) of Fabaceae in 1820. Secondary metabolites based on coumarin are widely distributed in numerous species of plants and microbes [15,17,18,19,20]. Coumarin derivatives can be classified into different categories based on their structural organization, possessing a wide range of pharmacological properties, including anticancer efficacy, especially against breast cancer [21]. Coumarin and its derivatives have been observed to be effective against different cancer types, including malignant and metastatic carcinomas in clinical trials, making it an optimum scaffold in anti-breast cancer drug development [22,23]. ADME studies confirmed that a very little amount of coumarin is excreted as unaltered form and is swiftly metabolized [24]. The substitution patterns on the coumarin core significantly influence the pharmacological potential of this nucleus, especially respective to breast cancer [25]. In the last three decades, inspired by the anticancer, and especially anti-breast cancer, properties of the coumarin nucleus, various research groups around the globe have tried to develop coumarin-based anticancer agents to eliminate drug resistance and toxicity-like issues of breast cancer management. This review is specifically focused on the development of coumarin-based small molecules that are effective against breast cancer and highlights their design strategy, structure–activity relationships, and molecular insights.

## 2. Naturally Occurring Coumarins with Anti-Breast Cancer Efficacy

Natural products have remained an ultimate source of medication for different diseases and disorders since antiquity, as evident from the remains of different civilizations around the globe. With advancements in modern analytical techniques, researchers have been successful in identifying the corresponding active principles from these natural medicinal sources (plants, animals, marine life, etc.) that have now become the basic leads, as well as established candidates, of modern clinical practice. Coumarins are one of these active principles/natural constituents that possess a wide array of pharmacological and therapeutic capabilities, and efforts are ongoing to identify novel coumarin derivatives from natural sources with varied pharmacological properties. Among the naturally occurring coumarins, numerous have been identified to possess anti-breast cancer efficacy, acting via distinct mechanisms, and have the potential to be drug adjuvants, as well as potential anti-breast cancer leads.

### 2.1. Simple Coumarins with Anti-Breast Cancer Potential

**Figure 1:**
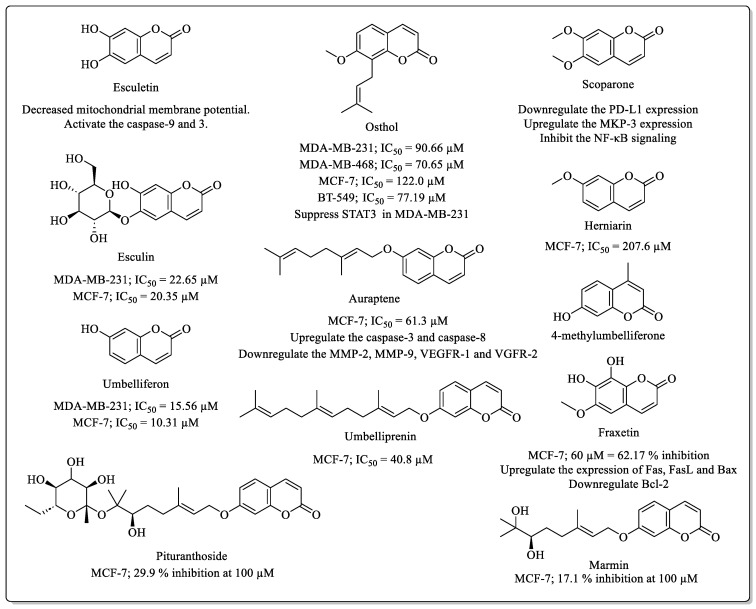
Simple coumarins from natural products with anti-breast cancer potential. A. 3-[4,5-Dimethylthiazol-2-yl]-2,5 diphenyl tetrazolium bromide (MTT) assay: osthole, auraptene pituranthoside, marmin, and herniarin were analysed after 72 h, while scoparone, esculin, umbelliferon, umbelliperin, and fraxetin were analysed after 48 h of incubation. B. Cell counting kit-8 (CCK-8) assay: 4-Methylumbelliferon was analysed after 24 h of incubation.

Numerous coumarin derivatives bearing native 2*H*-chromen-2-one cores (Figure 1) are widely explored for their breast cancer potential. Esculetin is a simple coumarin derivative found in the seeds of *Euphorbia lathyris* L. [26] and *Cichorium glandulosum* [27], which has been observed to induce calcium release from the endoplasmic reticulum in ZR-75-1 breast cancer cells, along with decreased mitochondrial membrane potential, released cytochrome C, and activated caspase-9 and 3. Esculetin induces cell cycle arrest at the G2/M phase of the cell cycle in ZR-75-1 breast cancer cells. Osthole is another simple coumarin found in *Cnidium monnieri* and *Angelica pubescens* [28], and it was effective against a range of breast cancer cells, including MDA-MB-231 (IC_50_ = 90.66 µM), BT-549 (IC_50_ = 77.19 µM), MDA-MB-468 (IC_50_ = 70.65 µM), and MCF-7 (IC_50_ = 122.0 µM). In triple-negative breast cancer cells (MDA-MB-231), osthole induced apoptosis by suppressing STAT3 and arresting the cell cycle at the G2/M phase, significantly decreased the tumour volume in MDA-MB-231 breast cancer cell xenografts in nude mice [29]. Scoparone, found in the leaves and stem of the *Artemisia capillaris*, downregulates the PD-L1 expression in dose-dependent manner and upregulates the MKP-3 expression [30]. Scoparone inhibits breast cancer cell survival by inhibiting the intercellular NF-κB signalling, which is associated with the *SNHG12/*miR-140-3p*/TRAF2* axis [31]. Herniarin, extracted from the flowers of *Matricaria chamomilla* L., displayed a cytotoxic effect on MCF-7 cells with an IC_50_ value of 207.6 µM and caused cell cycle arrest at the sub-G1 phase [32]. Esculin, which is a beta-glucose-6,7-dihydroxycoumarin found in *Aesculus hippocastanum* and *Aesculus californica*, displayed anti-breast cancer efficacy against MDA-MB-231 (IC_50_ = 22.65 µM) and MCF-7 (IC_50_ = 20.35 µM) cells [33]. 

Coumarins extracted from the umbelliferae family are widely popular for their diverse range of biological activities. Umbelliferone (7-hydroxycoumarin), a coumarin from the umbelliferae family plants, has been found to be effective against MDA-MB-231 (IC_50_ = 15.56 µM) and MCF-7 (IC_50_ = 10.31 µM) breast cancer cells and to arrest the cell cycle at the G2M phase in the MDA-MB-231 cells [34]. Similarly, 4-methylumbelliferone inhibited the proliferation of the T-47D and MDA-MB-231 breast cancer cells in a dose-dependent manner [35]. Moving forward with a similar pattern of structure, 7-prenyloxycoumarins are also well-studied for their biological efficacy. Auraptene is a simple coumarin analogue and is distributed among the citrus species of plants, such as lemon, grapefruit, and orange. Auraptene has been observed to decrease the cell viability, migration, and angiogenesis in MCF-7 cells (IC_50_ = 61.3 µM) and to induce apoptosis via upregulating the caspase-3 and caspase-8 and downregulating the MMP-2, MMP-9, VEGFR-1, and VGFR-2 genes [36]. Similarly, umbelliprenin, another 7-prenyloxycoumarin, is effective against MCF-7 cells, with an IC_50_ value of 40.8 µM. Fraxetin, found in the bark of *Fraxinus rhynchophylla* and *Fraxinus bungeana*, inhibits the proliferation of MCF-7 cells (62.17% at 60 µM) and has been observed to induce apoptosis by upregulating the expression of *Fas*, *FasL*, and *Bax*, while downregulating Bcl-2 [37].

### 2.2. Furanocoumarins Coumarins with Anti-Breast Cancer Potential

**Figure 2:**
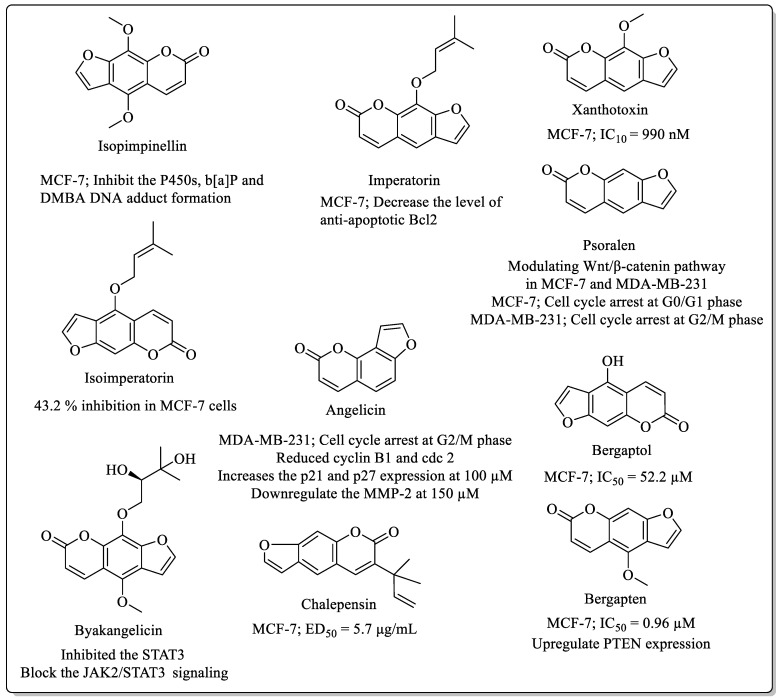
Furanocoumarins from natural products with anti-breast cancer potential. MTT assay: bergapten and chalepensin were analysed after 5 and 7 days of incubation. Isopimpinellin, isoimperatorin, and bergapten were analysed after 72 h of incubation. Psorelin and angelicin were analysed after 48 h of incubation. Bergaptol was analysed after 24 h of incubation.

Furanocoumarins (Figure 2) are an elite class of coumarins that are widely explored for their broad range of biological activities, including anticancer potential. Numerous furanocoumarins are observed to possess anti-breast cancer potential with their distinct mechanisms. Isopimpinellin, found in the *Deverra tortuosa* plant, showed inhibitory properties against MCF-7 cells and was found to inhibit P450s (including 1A1, 1B1, 1A2 and 3A4). Isopimpinellin was also responsible for the inhibition of b[a]P and DMBA-DNA adduct formation in MCF-7 cells [38,39]. Imperatorin, found in *Cnidium monnieri*, was also observed to decrease the proliferation of the MCF-7 breast cancer cells upon treatment, by decreasing the level of anti-apoptotic Bcl2. Xanthotoxin displayed an IC_10_ value of 990 nM [40] and Isoimperatorin, another furanocoumarin derived from *Deverra tortusa*, exhibited 43.2% inhibition of MCF-7 cells [38].

Psoralen, extracted from *Psoralea corylifolia*, inhibited the proliferation of MCF-7 and MDA-MB-231 cells in a dose-dependent manner, caused cell cycle arrest in MCF-7 cells at the G0/G1 phase and at the G2/M phase in MDA-MB-231 cells, and modulated the Wnt/β-catenin pathway [41]. Angelicin, extracted from the fruit of *Psoralea corylifolia* L. and *Angelica archangelica*, inhibited cell proliferation and arrested the cell cycle at the G2/M phase in MDA-MB-231 cells. Angelicin reduced cyclin B1 and cdc2 and increased the p21 and p27 expression at the concentration of 100 µM. At the concentration of 150 µM, it inhibited the migration and invasion of MDA-MB-231 cells and partially downregulated the MMP-2 protein levels [42]. Byakangelicin, extracted from the root of *Angelica dahurica*, inhibited the STAT3 transcriptional activity and blocked the JAK2/STAT3 signalling in a dose-dependent manner. Chalepensin, extracted from the *Stauranthus perforates*, exhibited the ED_50_ value of 5.7 μg/mL against MCF-7 cells [43]. Bergaptol, a natural furanocoumarin derivative generally found in lemons, exhibited inhibitory potential against MCF-7 cells, with an IC_50_ value of 52.2 µM [44]. Similarly, bergapten, another furanocoumarin, showed inhibitory efficacy against MCF-7 cells, with an IC_50_ value of 0.96 µM, and upregulated PTEN expression [40].

### 2.3. Sesquiterpene Coumarins with Anti-Breast Cancer Potential

**Figure 3:**
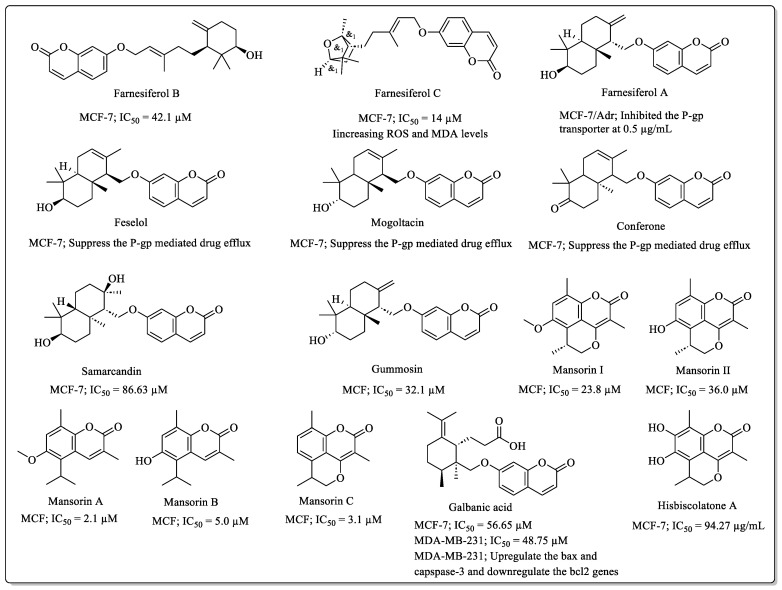
Sesquiterpene coumarins from natural products with anti-breast cancer potential. Alamar blue assay: Farnesiferol B and samarcandin were analysed after 24 and 72 h of incubation respectively. MTT assay: Galbanic acid was analysed after 24 h of incubation. Farnesiferol B and Hisbiscolatone A were analysed after 72 h of incubation. Sulforhodamine B (SRB) assay: all mansorins were analysed after 72 h of incubation.

Sesquiterpene coumarins (Figure 3) are a distinct class of coumarin derivatives widely spread among different plant species, especially among *Ferula* species, and widely explored for their therapeutic properties in the last three decades. Farnesiferol B, a sesquiterpene coumarin extracted from *Ferula asafoetida*, showed efficacy against MCF-7 cells (IC_50_ = 42.1 µM), along with higher selectivity toward breast cancer cells over normal fibroblast cells (NIH: IC_50_ > 100 µM) [45]. Farnesiferol C exhibited anti-breast cancer efficacy against MCF-7 cells (IC_50_ = 14 µM) and induced apoptosis by increasing the ROS and MDA levels [46]. Farnesiferol A was effective against MCF-7/Adr cells and inhibited the P-gp transporter at the concentration level of 0.5 µg/mL, being more potent than the standard verapamil [47]. Feselol, obtained from the *Ferula badrakema* and *Ferula gummosa* fruits, suppressed the P-gp mediated drug efflux in the highly resistant MCF-7 breast cancer cells [48]. Mogoltacin is another sesquiterpene from the fruits of *Ferula gummosa* that suppressed the P-glycoprotein-mediated MDR in MCF-7 cells [48]. Conferone, extracted from the fruits and roots of *Ferula* species, was also found to suppress the P-gp-mediated drug efflux in MCF-7 cells. Similarly, samarcandin, extracted from *Ferula asafoetida*, displayed efficacy against MCF-7 cells (IC_50_ = 86.63 µM) [49]. Gummosin, found in the *Ferula asafoetida*, was also effective against MCF-7 cells (IC_50_ = 32.1 µM) [45].

Mansonones, the sesquiterpene-derived ortho-naphthoquinones, are a group of coumarins distributed among the Hibiscus, Mansonia, and Thespesia genera. Some mansorins from the *Mansonia gagei* plant, including Mansorin A (IC_50_ = 2.1 µM), Mansorin B (IC_50_ = 5.0 µM), Mansorin C (IC_50_ = 3.1 µM), Mansorin I (IC_50_ = 23.8 µM), and Mansorin II (IC_50_ = 36.0 µM), were found to be effective against MCF-7 cells. Galbanic acid, a sesquiterpene coumarin derivative, and a terpene lactone are widely distributed among the *Ferula* species and exhibited various biological activities. Galbanic acid was effective against MCF-7 (IC_50_ = 56.65 µM) and MDA-MB-231 (IC_50_ = 48.75 µM) breast cancer cell lines and induced apoptosis in MDA-MB-231 cells by the upregulation of bax and capspase-3 and the downregulation of the bcl2 genes [50]. Similarly, Hisbiscolatone A, extracted from the stems of *Helicteres hirsute*, showed efficacy against MCF-7 cells, with an IC_50_ value of 94.27 µg/mL [51]. 

### 2.4. Miscellaneous Naturally Occurring Coumarins with Anti-Breast Cancer Potential

**Figure 4:**
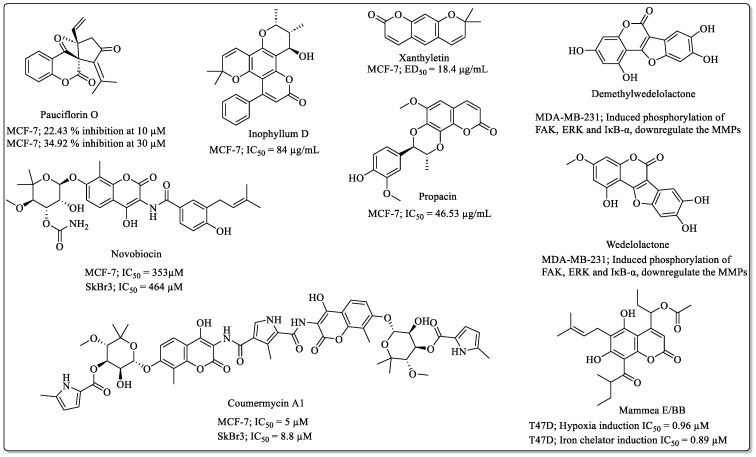
Miscellaneous coumarins from natural products with anti-breast cancer potential. MTT assay: Xanthyletin was analysed after 7 days of incubation. Novobiocin and Inophyllum D were analysed after 24 h of incubation. Pauciflorin O was analysed after 72 h of incubation. Sulforhodamine B (SRB) assay: Mammea E/BB was analysed after 48 h of incubation.

The Monoterpenoid class of coumarins also exhibited an interesting range of pharmacological properties and was evaluated for anti-breast cancer efficacy too. Pauciflorin O, a white amorphous solid coumarin derivative isolated from *Centrapalus pauciflorus*, showed 22.43% and 34.92% inhibition of MCF-7 cells at the concentration levels of 10 µM and 30 µM, respectively. However, the compound was found inactive against MDA-MB-231 [52]. Marmin and pituranthoside, found in *Deverra tortuosa*, inhibited the growth of MCF-7 cells by 17.1% and 29.9% at the concentration level of 100 μM [38]. Inophyllum D is an angular-type natural coumarin derivative from the *Calophyllum symingtonianum* active against the MCF-7 breast cancer cells, with an IC_50_ value of 84 µg/mL [53]. Xanthyletin, a pyranocouamrin, exhibited the ED_50_ value of 18.4 μg/mL against MCF-7 cells [43]. Ammirin, another coumarin derivative, showed efficacy against breast cancer cells, with an ED_50_ value of 46.9 μg/mL [43]. Chalepin, from *Ruta angustifolia*, displayed an IC_50_ value of 8.5 μg/mL against MCF-7 cells and 19.8 μg/mL against the MDA-MB-231 cells. It induced apoptosis via activation of the P38 MAPK signalling pathway [54]. Propacin from *Helicteres hirsute* exhibited an IC_50_ value of 46.53 µg/mL against MCF-7 cells [51]. Novobiocin from *Streptomyces spheroids* and *Streptomyces niveus* exhibited IC_50_ values of 353 and 464 µM against the MCF-7 and SkBr3 breast cancer cells [55], inhibited their migration, and suppressed the SMYD3 pathway [56]. Coumermycin A1, an aminocoumarin derivative, exhibited IC_50_ values of 5 µM and 8.8 µM against the MCF-7 and SkBr3 breast cancer cell lines [55]. Wedelolactone, extracted from *Wedelia calendulacea*, suppressed the growth and invasion of the MDA-MB-231 cells, as well as induced the phosphorylation of FAK, ERK, and IκB-α, which leads to the downregulation of MMPs [57]. Demethylwedelolactone, isolated from *Eclipta alba*, inhibited the growth and invasion of the MDA-MB-231 cells and downregulated MMPs by phosphorylating FAK, ERK, and IκB-α [57]. The Mammea E/BB, extracted from *Mammea americana* L., inhibited the hypoxia-induced and iron chelator-induced HIF-1 activation in breast tumour T47D cells, with IC_50_ values of 0.96 and 0.89 µM. Mammea E/BB also suppressed the VEGF in T47D cells at the concentration of 5 µM and inhibited the migration of the MDA-MB-231 cells at the concentration level of 20 µM (Figure 4) [58].

## 3. Synthetic Coumarin-Inspired Derivatives with Anti-Breast Cancer Efficacy

Inspired by the anti-breast cancer potential of various naturally occurring coumarin derivatives, various coumarin-containing synthetic derivatives have been developed by different research groups around the globe in last three decades and provided potential leads for advancing the anti-breast cancer drug research.

### 3.1. Coumarin and Piperazine Conjugates as Anti-Breast Cancer Agents

**Figure 5:**
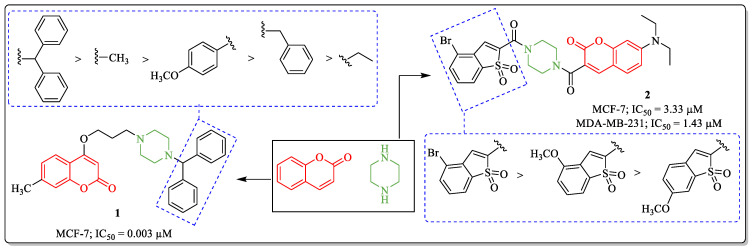
Coumarin and piperazine conjugates with anti-breast cancer efficacy. MTT assay: compound **1** was analysed after 24 h of incubation, compound **2** was analysed after 48 h of incubation.

Piperazine is a heterocyclic six-membered ring containing two nitrogen atoms on opposite sides. The scaffold itself is active in various biological activities and also serves as a linker molecule in the field of medicinal chemistry [59]. The presence of nitrogen in any molecule plays an essential role in its bioavailability and enhances its solubility [60]. This nitrogen-containing moiety is under continuous exploration by various research groups for its potential in combating numerous diseases. Inspired by the biological potential of coumarin and piperazine, Patel et al. designed and synthesized a series of 4-hydroxy-7-methylcoumarin derivatives and evaluated their anticancer efficacy. The results highlighted compound **1** as the most active anti-breast cancer agent with an IC_50_ value of 0.003 µM against the MCF-7 breast cancer cell line. The structure–activity relationship suggested that the substitution of biphenyl rings on the piperazine with the methyl bridge was the most desirable for anti-breast cancer activity of the structure, and, at the same time, the replacement of the biphenyl rings with the methyl, 4-methoxyphenyl, benzyl, or ethyl groups resulted in a decrease in activity [61]. An *N*,*N*-diethyl-7-aminocoumarin fluorophore with benzo [*b*]thiophene 1,1-dioxide derivative (compound **2**), prepared by Cai et al., showed efficacy against MCF-7 and MDA-MB-231 cells, with IC_50_ values of 3.3 µM and 1.43 µM, respectively. The structure–activity pattern revealed the desirability of the substitution of the benzothiophene ring for the activity of the compound. At the same time, the introduction of bromine at the fourth position of the benzothiophene elevates the activity profile, while the replacement of bromine with the methoxy group leads to a decrease in the anti-breast cancer potential of the structure (Figure 5) [62]. 

### 3.2. Coumarin and Piperidine Conjugates as Anti-Breast Cancer Agents

**Figure 6:**
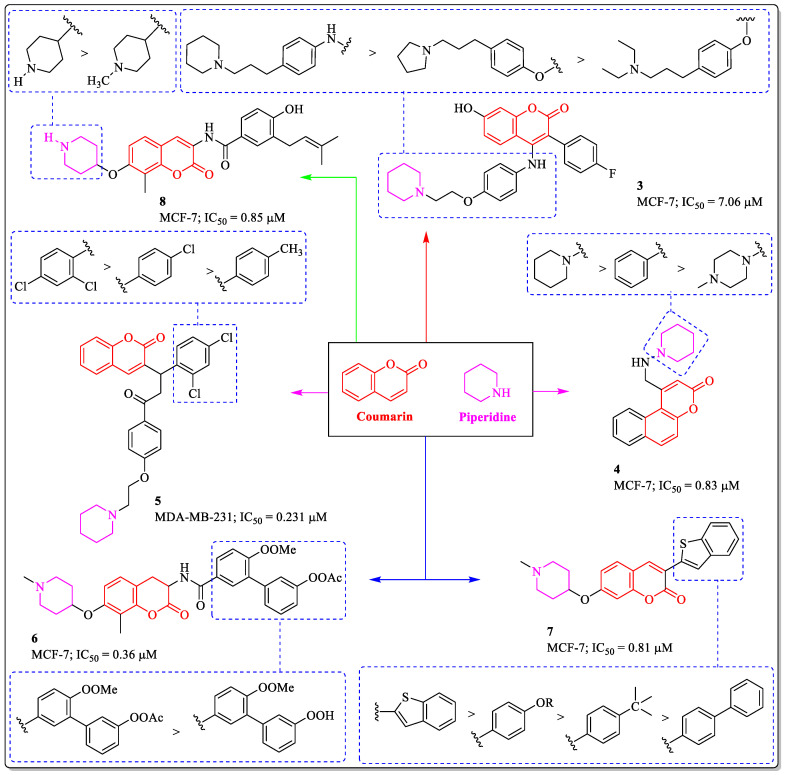
Coumarin and piperidine conjugates with anti-breast cancer efficacy. MTT assay: compounds **3** and **4** were analysed after 48 h of incubation. Sulforhodamine B (SRB) assay: compound **5** was analysed after 72 h of incubation. MTS/PMS cell proliferation assay: compound **8** was analysed after 72 h of incubation.

Another heterocyclic organic compound, piperidine, containing an amine group, is extensively used as a reagent and building block in medicinal chemistry [63]. Piperidine-appended anticancer drugs have already delivered their potential role in cancer treatment [64]. The multifunctional directed ligands (MTDL) approach can improve the efficacy and minimize the side effects. Inspired by this, Luo et al. designed and synthesized a series of 3-aryl-4-anilino/aryloxy-2*H*-chromen-2-one analogues and assessed their anticancer potential. The results of the assay suggested that compound **3** emerged as the most potent candidate, with an IC_50_ value of 7.06 µM against MCF-7 breast cancer cell line. The structure–activity relationship revealed that the substitution of the piperidine ring was beneficial for the activity, while replacing the ring with the pyrrolidine or diethyl amine resulted in a loss of activity. The presence of an amine group between the phenyl ring and coumarin was essential for the activity of the compound [65]. A 3*H*-benzo[f]chromen-3-one derivative, compound **4**, developed by Soni et al., showed efficacy against MCF-7 cells, with an IC_50_ value of 0.83 µM. The analysis of the structure–activity pattern revealed the importance of the piperidine ring linked to the coumarin in the anti-breast cancer activity of the structure, whereas the replacement of the piperidine ring with phenyl or piperazine led to a decrease in potency [66]. Similarly, the 3-(3-oxosubstitutedphenyl-3-)4-(2-(piperidinyl)ethoxy)phenyl)propyl)-2*H*-chromen-2-one derivative (compound **5**) developed by Dube et al. showed efficacy against MCF-7 cells, with an IC_50_ value of 0.231 µM. The structure–activity pattern here revealed that the substitution of a chlorine atom at the *ortho* and *para* position of the phenyl ring is necessary for the activity of the compound, while either the removal of chlorine from the *ortho* position or the introduction of a methyl group at the *para* position resulted in a significant decrease in potency [67]. Compound **6**, a novobiocin analogue developed by Zhao et al., showed efficacy against MCF-7 cells, with an IC_50_ value of 0.36 µM. The structure–activity pattern suggested that the replacement of ethaneperoxoate with an hydroxyperoxy group on the biphenyl nucleus significantly reduced the anticancer potential of the structure [68]. Similarly, a 3-arylcoumarin derivative, compound **7**, also showed efficacy against MCF-7 cells, with an IC_50_ value of 0.18 µM. The structure–activity pattern revealed that the unsubstituted benzothiophene ring was the most suitable for the activity, while the replacement with the substituted phenyl ring or biphenyl rings led to a decrease in potential [69]. Zhao et al. designed and synthesized novobiocin analogues as potent anticancer agents against breast cancer cells. Among them, compound **8** was found to be the most potent anti-breast cancer agent, with an IC_50_ value of 0.85 µM against the MCF-7 cell line. The structure–activity relationship suggested that the compound with piperidine was found to be the most potent anti-breast cancer compound, while the potency of the compound decreases with the substitution of the methyl group linked to the piperidine ring (Figure 6) [70].

### 3.3. Bis-Coumarin Derivatives as Anti-Breast Cancer Agents

**Figure 7:**
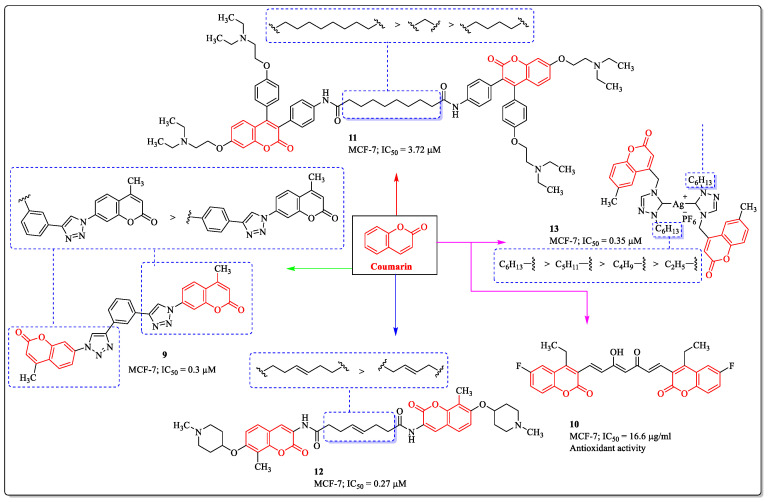
Bis-coumarin derivatives with anti-breast cancer efficacy. MTT assay: compounds **11** and **13** were analysed after 48 h of incubation. Compounds **9**, **10**, and **12** were analysed after 72 h of incubation.

Bis-coumarin derivatives are a parallel class of bioactive compounds that have a distinguished place in the field of medicinal chemistry and possess a broad range of biological properties, including anti-breast cancer efficacy [71]. A bis-coumarin derivative, compound **9**, developed by Pršir et al., was effective against MCF-7 cells, with an IC_50_ value of 0.3 µM. The analysis of the structure–activity relationship conferred that the 1,3-bis(coumarin-triazolyl) derivative exhibited stronger activity compared to the 1,4-disubstituted structural isomer. A change in substitution from the third to the fourth position led to a decrease in activity [72]. Inspired by the pharmacological characteristics and broad-spectrum medicinal properties of curcumin and based on the structural features of the curcumin, Oglah et al. designed and synthesized curcumin analogues and evaluated their anti-breast cancer activity against breast cancer cell lines. Among them, compound **10** was found to be the most potent anti-breast cancer agent, with an IC_50_ value of 16.6 µg/mL against MCF-7 breast cancer cells [73]. Inspired by the dimeric compounds and their potential against various cancers, Zhu et al. designed and synthesized triphenylethylene-coumarin hybrids with two amino side chains and screened for their anticancer activity. Among them, compound **11** was the most effective against MCF-7 cells with an IC_50_ value of 3.72 µM. The structure–activity relationship suggests the influence of the linker between the dimeric compounds on their activity. An eight-carbon linker was observed to be the most effective. A decrease or an increase in the chain length greatly influences the anti-breast cancer potential of the structure [74]. A Coumermycin A1 analogue, compound **12**, developed by Kusuma et al., showed effectiveness toward MCF-7 cells, with an IC_50_ value of 0.27 µM. The structure–activity relationship here again suggests the importance of the linker. A length of up to six carbons was beneficial for its activity, while a decrease in the chain length led to a decrease in its potential [75]. A coumarin-substituted 1,2,4-triazole-derived silver (I) and gold (I) complex, compound **13**, showed activity toward MCF-7 cells with an IC_50_ value of 0.35 µM. The structure–activity relationship suggested that the substitution of alkyl chain up to the six carbons on both triazole rings was best suited for its activity, while a decrease in the chain length led to a reduction in activity (Figure 7) [76].

### 3.4. Indole–Coumarin Derivatives as Anti-Breast Cancer Agents

**Figure 8:**
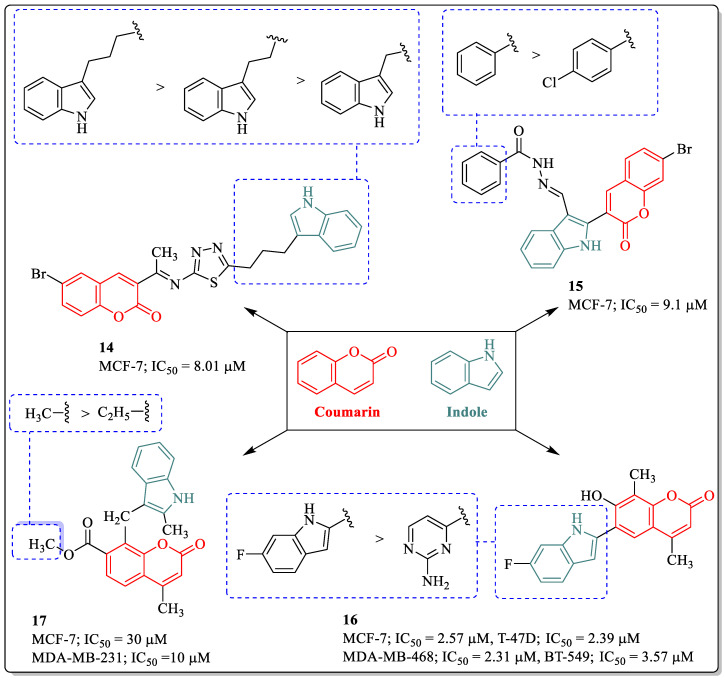
Indole–coumarin derivatives with anti-breast cancer efficacy. MTT assay: compounds **14**, **15**, and **17** were analysed after 48 h of incubation. Sulforhodamine B (SRB) assay: compound **16** was analysed after 48 h of incubation.

Indole is a heterocyclic compound found in various plant sources, and the synthetic derivatives of indole have displayed biological potential, including anticancer activity. The available drugs with an indole ring in the market have already established their importance [77]. Inspired by the pharmacological efficacy of multifunctional directed ligands (MTDLs) developed through molecular hybridization, Kamath et al. designed and synthesized indole–coumarin–thiadiazole hybrids and evaluated them for their anti-breast cancer activity. The results of the assay suggested that compound **14**, with an IC_50_ value of 8.01 µM against MCF-7 cells, was found to be the most potent anti-breast cancer agent. The structure–activity relationship demonstrated that the substitution of an indole linked to the thiadiazole was essential for activity, while the linker between the thiadiazole and indole had a great effect on the activity profile. A length up to three carbons was optimum for the activity of the compound, whereas any change in the length led to reduced activity [78]. Similarly, an indole–coumarin hybrid Schiff base, compound **15**, was found to be efficacious against MCF-7 cells with an IC_50_ value of 9.1 µM. The substitution of the phenyl ring linked to an amide group was beneficial for the activity of the compound, while the introduction of an electronegative or electropositive group on the phenyl ring leads to a decrease in activity [79]. 6-Heteroaryl coumarin, compound **16**, designed and synthesized by Galayev et al., was found to be the most potent anti-breast cancer agent, with IC_50_ values of 2.57 µM, 2.39 µM, 2.31 µM, and 3.57 µM against the MCF-7, T-47D, MDA-MB-231, and BT-549 cell lines. The structure–activity pattern suggested that the compound with fluorine attached to the C6 position of the indole moiety of coumarin was found to be most potent, while the activity decreased in the presence of 2-amino pyrimidine moieties [80]. Mazzei et al. designed and synthesized unsymmetrical-methylene derivatives of indole and evaluated their anticancer potential against breast cancer cell lines. Among them, compound **17** was found to be most effective against MCF-7 cells, with an IC_50_ value of 30 µM. An acetoxy group at the seventh position of coumarin was found to be most suitable for the activity in compound **17**. The replacement of the methyl group with an ethyl group led to a decrease in the anti-breast cancer potential of the structure (Figure 8) [81].

### 3.5. Coumarin and Isatin Conjugates as Anti-Breast Cancer Agents

**Figure 9:**
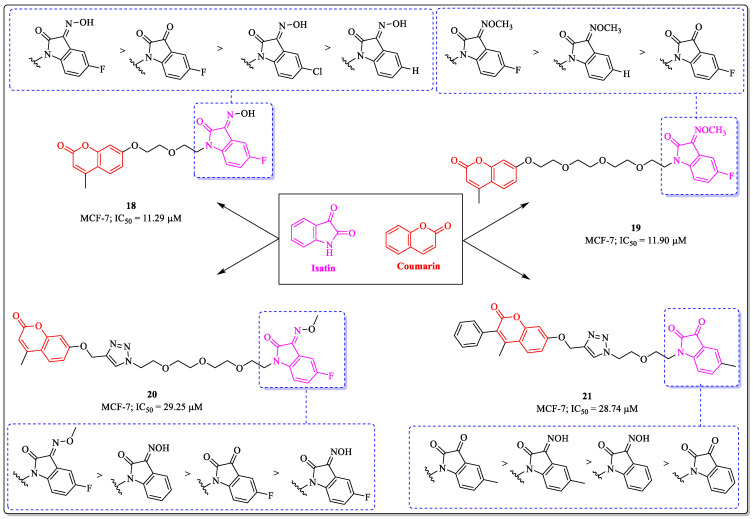
Isatin–coumarin conjugates with anti-breast cancer efficacy. Sulforhodamine B (SRB) assay: compounds **18**–**21** were analysed after 5 days of incubation.

Isatin is a privileged scaffold in medicinal chemistry and possess various pharmacological properties. Derivatives of isatin are being used in clinical trials, as well as in clinical practice, to tackle various types of cancers. The structure–activity relationship studies revealed that a linker between isatin and coumarin can influence the activity profile of the compounds. Inspired by this, Fan et al. designed and synthesized isatin and coumarin hybrids. The compounds were evaluated for their anticancer activity. Among them, compound **18** was found to be the most potent anti-breast cancer candidate, with an IC_50_ value of 11.29 µM, against MCF-7 cells. The structure–activity relationship suggested that the substitution of fluorine at the fifth position of isatin was beneficial for the activity of the compound, while the replacement with a chlorine atom decreased the activity of the compound. The introduction of a NOH group at the third position of the isatin led to a decrease in activity [82]. Similarly, Xu et al. introduced a glycol fragment and synthesized glycol-tethered isatin–coumarin hybrids, which were evaluated for anticancer activity against breast cancer cell lines. Out of them, compound **19** was most effective against MCF-7 cells, with an IC_50_ value of 11.9 µM. The substitution of fluorine at the fifth position of isatin was observed to be essential for the activity of the compound, while the replacement of fluorine with hydrogen resulted in a decrease in activity. The introduction of methoxyamine at the third position of the isatin nucleus was desirable for its activity, while the replacement of methoxyamine with carbonyl resulted in decreased activity [83]. Xu et al. designed and synthesized tetra-ethylene glycol-tethered isatin triazole–coumarin hybrids and compound **20**, among them, was found to be most potent against MCF-7 cells, with an IC_50_ value of 29.25 µM. The substitution of fluorine at the fifth position of isatin was desirable for the activity of the compound, while the replacement of fluorine with hydrogen resulted in a decrease in activity. The introduction of methoxyamine at the third position of isatin was beneficial for its activity, and, again, the replacement of methoxyamine with carbonyl resulted in a decrease in activity [84]. Diao et al. synthesized diethylene glycol-tethered isatin–coumarin hybrids and evaluated their anticancer activity. The results of the assay suggested that compound **21**, with an IC_50_ value of 28.74 µM against the MCF-7 cell line, was the most potent. An unsubstituted isatin ring is desirable for the activity in this structure, whereas the introduction of any substitution on the isatin ring leads to a decrease in the potential of the compound (Figure 9) [85].

### 3.6. Coumarin and Chalcone Conjugates as Anti-Breast Cancer Agents

**Figure 10:**
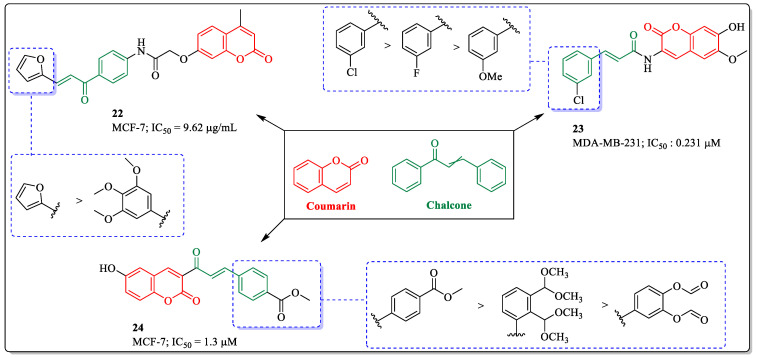
Coumarin–chalcone conjugates with anti-breast cancer efficacy. MTT assay: compounds **23** and **24** were analysed after 72 h of incubation. Sulforhodamine B (SRB) assay: compound **22** was analysed after 48 h of incubation.

Chalcones are the precursors of flavonoids and isoflavonoids and are widely present in nature. Among all the flavonoids, chalcones are mostly used to investigate broad-spectrum biological activities. The chalcone fragment has a promising anticancer profile, being capable of inducing apoptosis and of collapsing the mitochondrial membrane. The literature also suggested that this fragment has other anticancer mechanisms, such as tubulin polymerization prevention via binding the colchicine binding site [86,87]. Inspired by the strategy of molecular hybridization, El-Sherief et al. designed and synthesized a series of coumarin–chalcone derivatives and evaluated their anticancer activity against breast cancer cells. Among them, compound **22** was found to be most effective against MCF-7 cells, with an IC_50_ value of 9.62 µg/mL. The substitution of the furan ring linked to the phenyl ring resulted in an increase in activity, while the replacement of furan with the tri-methoxy-substituted phenyl ring decreased the activity of the compound [88]. A scopoletin–cinnamic hybrid, compound **23**, developed by Li et al., showed efficacy against MCF-7 cells, with an IC_50_ value of 0.231 µM. The substitution of a chlorine atom at the *meta* position of the benzyl ring was found to be beneficial for the activity of the compound, while the substitution of fluorine or a methoxy group at the *meta* position significantly decreased its activity [89]. The coumarin derivative **24**, developed by Molaverdi et al., showed efficacy against MCF-7 cells, with an IC_50_ value of 1.3 µM. In this compound, the substitution of methyl acetate at the *para* position of the benzyl ring was best suited for the activity of the compound, while the substitution of a methoxy group or methyl formate led to a decrease in its activity profile (Figure 10) [90].

### 3.7. Coumarin and Quinoline Hybrids as Anti-Breast Cancer Agents

**Figure 11:**
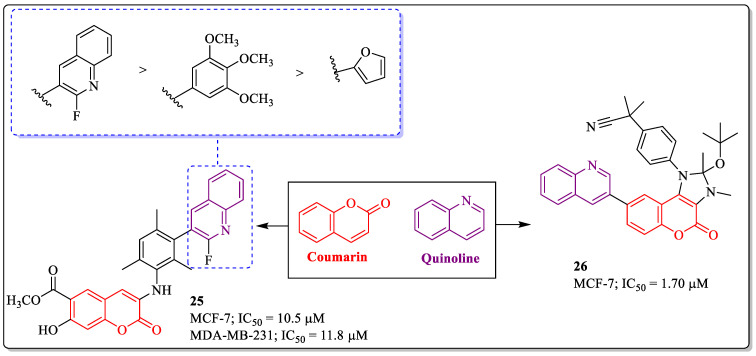
Coumarin–chalcone hybrids with anti-breast cancer efficacy. MTT assay: compounds **25** and **26** were analysed after 72 h of incubation.

Quinoline is an aromatic heterocyclic organic nucleus that has the ability to target cancer-specific signals or enzymatic routes [91]. Derivatives of quinoline have the potential to inhibit tyrosine kinase, topoisomerase, tubulin polymerization, and DHODH kinase [92,93,94,95]. Inspired by the biological potential of quinoline and coumarin, Lipeeva et al. designed and synthesized a series of 3-(*N*-substituted) aminocoumarin derivatives. Among them, compound **25** was most effective against MCF-7 cells, with an IC_50_ value of 10.5 µM. The substitution of quinoline rings linked to the phenyl ring was best suited for the activity of the compound. The replacement of quinoline rings with the substituted phenyl or furan ring resulted in a decrease in its efficacy [96]. Similarly, a 2,3-dihydrochromeno[3,4-*d*]imidazol-4(1*H*)-one derivative (compound **26**) developed by Han et al. showed efficacy against MCF-7 cells, with an IC_50_ value of 1.70 µM (Figure 11) [97].

### 3.8. Coumarin-Appended Alkyl Chains as Anti-Breast Cancer Agents

**Figure 12:**
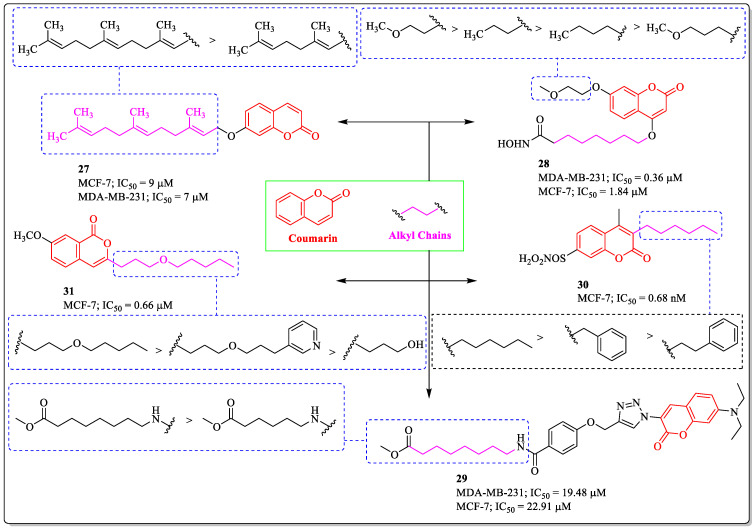
Coumarin derivatives bearing alkyl chains with anti-breast efficacy. MTT assay: compounds **27** and **29** were analysed after 48 h of incubation. Sulforhodamine B (SRB) assay: compounds **28** and **31** were analysed after 48 h of incubation.

In the past few decades, it has been observed that alkyl chains at the end of the molecules can synergize the activity of the compound due to their flexibility towards the receptors. Gkionis et al. designed and synthesized a series of bioinspired synthetic alkoxy–coumarin derivatives. The compounds were subjected to the evaluation of anticancer activity. Among them, compound **27** was the most effective, with an IC_50_ value of 9 µM against MCF-7 cells. The length of the chain linked to the coumarin has a significant influence on the activity of the compound: with the increase in the chain length, activity increases [98]. Similarly, coumarin-based hydroxamate (compound **28**) developed by Zhao et al. showed efficacy against MCF-7 cells, with an IC_50_ value of 1.84 µM. The presence of a terminal methoxy group on a two-carbon alkyl chain at the seventh position of coumarin was found to be most effective for enhancing activity. Any increase in the chain length or substitution of the terminal methoxy with a methyl group resulted in decreased activity [99]. A series of santacruzamate A analogues were synthesized by Andrade et al. and investigated for their anticancer potential. Among them, compound **29** was found to be the most potent anti-breast cancer agent, with an IC_50_ value of 19.48 µM against the MDA-MDB-231 breast cancer cells. The structure–activity relationship suggested that the substitution of a longer carbon chain linked to the phenyl ring via an amide linkage was beneficial for the activity of the compound, while decreases in the length of the chain led to decreased activity [100]. Ganeshapillai et al. designed and synthesized C-3- and C-4-substituted bicyclic coumarin sulfamate (compound **30**), which was effective against MCF-7 cells, with an IC_50_ value of 0.68 µM. The substitution of the alkyl chain on the coumarin ring is desirable for the activity, while the replacement of the alkyl chain with the benzyl ring resulted in decreased activity [101]. Guimarães et al. synthesized a series of isocoumarin and 3,4-dihydrocoumarin derivatives that were effective against MCF-7 cells, and compound **31** was most potent, with an IC_50_ value of 0.66 µM. A 3-(Pentyloxy) propyl group substituted on the coumarin ring was most beneficial for the activity of the compound, while the introduction of a pyridine ring or hydroxy group at the end of the chain led to decreased efficacy (Figure 12) [102].

### 3.9. Coumarin and Pyrimidine Hybrids as Anti-Breast Cancer Agents

**Figure 13:**
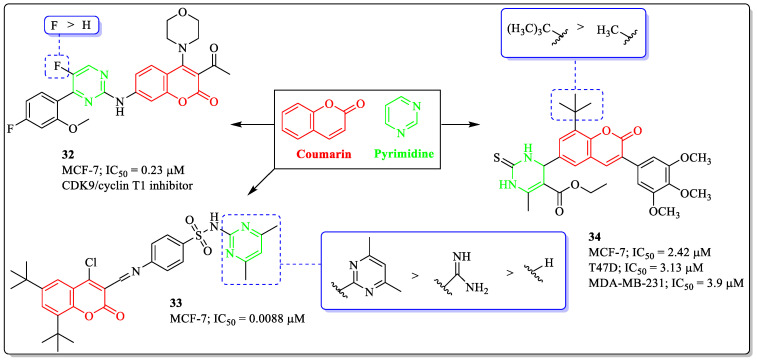
Coumarin–pyrimidine hybrids with anti-breast cancer efficacy. MTT assay: compounds **33** and **34** were analysed after 48 h of incubation. CellTiter-Glo^®^ Reagent assay: compound **32** was analysed after 72 h of incubation.

In silico studies suggest that pyrimidine moiety forms hydrogen bond interactions with the receptor with the help of its nitrogen atoms. Dihydro-pyrimidinone was found to be a cell-permeable molecule and causes mitotic arrest by blocking the bipolar mitotic spindle in mammalian cells [103,104]. These studies clearly indicated the importance of pyrimidine in the anticancer drug development. Taking lead from the pharmacological properties of coumarin and pyrimidine, Xu et al. designed and synthesized coumarin derivatives and evaluated their anticancer potential. Among the whole series, compound **32** was effective against MCF-7 cells, with an IC_50_ value of 0.23 µM. The substitution of fluorine at the fifth position of the pyrimidine ring was best suited for the activity of the compound, whereas replacement with hydrogen led to decreased activity [105]. A coumarin-containing sulfonamide derivative (compound **33**) developed by Wang et al. showed efficacy against MCF-7 cells, with an IC_50_ value of 0.0088 µM. A methyl group on the pyrimidine ring was best suited for the activity of the compound, while the replacement of the pyrimidine ring with *N*-carbamimidoyl or no substitution led to decreased activity[106]. A coumarin–monastrol hybrid (compound **34**) developed by Sashidhara et al. showed anti-breast cancer activity, with IC_50_ values of 2.42 µM, 3.13 µM, and 3.9 µM against the MCF-7, T-47D, and MDA-MB-231 cells. The substitution of the tertiary butyl group on the coumarin ring was beneficial for the activity of the compound, while the replacement of the tertiary butyl group with a methyl group led to decreased activity (Figure 13) [107].

### 3.10. Coumarin and Pyridine Hybrids as Anti-Breast Cancer Agents

**Figure 14:**
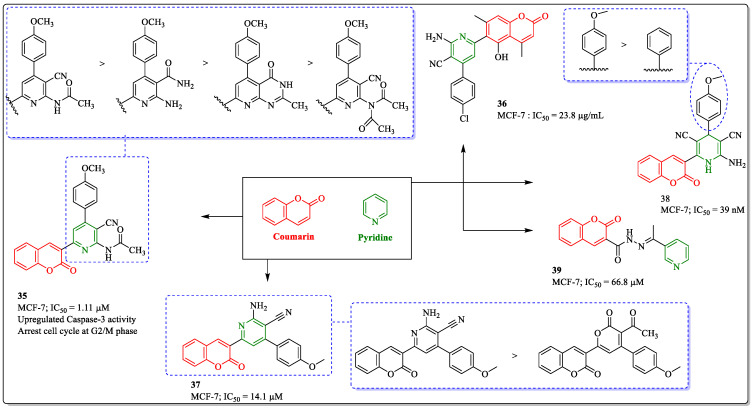
Coumarin–pyridine hybrids with anti-breast cancer efficacy. MTT assay: compounds **35**–**38** were analysed after 24 h of incubation.

Pyridine and fused pyridines are believed to have various biological activities [108]. A literature survey reported the importance and usefulness of this scaffold against cancer [109]. A number of molecules have been developed based on pyridine in the past decades, with potential anti-breast cancer activities [110,111,112]. Inspired by the coumarin and pyridine hybridization approach, Fayed et al. designed and synthesized a series of coumarin derivatives, and their anticancer activity was tested on various cell lines. Among them, compound **35**, with an IC_50_ value of 1.1 µM against the MCF-7 breast cancer cell line, was the most potent agent. The structure–activity relationship suggested that substituting the cyano group at the third position and the amino-acteyl group at the second position was beneficial for the activity of the compound, whereas replacing the cyano group with any other substituent led to decreased activity. When introducing a pyridopyrimidine ring instead of the pyridine ring, the activity also diminished [113]. Hassan et al. developed substituted and fused coumarin derivatives and evaluated them for their anticancer activity. Among them, compound **36** was found to be most potent, with an IC_50_ value of 23.8 µg/mL against MCF-7 breast cancer cells [114]. A series of coumarin derivatives were synthesized by El-Naggar et al. and evaluated for their anticancer activity. Among them, compound **37** was most effective against MCF-7 cells, with an IC_50_ value of 14.1 µM. The presence of a pyridine ring liked to the coumarin ring was essential for the activity of the compound. The substitution of the amino and cyano group at the second and third position significantly elevated the activity profile [115]. A benzopyran-2-one derivative (compound **38**), developed by Mohareb et al., showed efficacy against MCF-7 cells, with an IC_50_ value of 39 nM. The substitution of the methoxy group at the *para* position of the phenyl ring significantly elevated the activity profile, while no substitution decreased the potential of the compound [116]. Similarly, a hydrazide–hydrazone coumarin derivative (compound **39**), developed by Mohareb et al., showed effectiveness against MCF-7 cells, with an IC_50_ value of 66.8 µM (Figure 14) [117].

### 3.11. Coumarins Clubbed with Aloe Emodin, Harmine, and Ergosterol as Anti-Breast Cancer Agents

**Figure 15:**
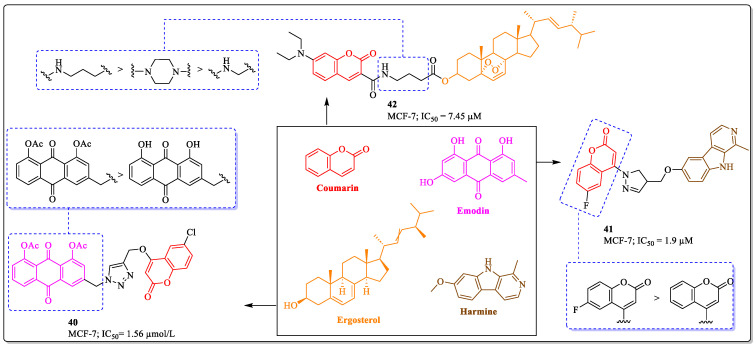
Coumarins clubbed with aloe emodin, harmine, and ergosterol derivatives, with anti-breast cancer efficacy. MTT assay: compounds **40** and **42** were analysed after 48 h, while **41** was analysed after 72 h of incubation.

The natural anthraquinone aloe emodin is obtained from *Rheum palmatum* L. and *Aloe vera* L. Aloe emodin displays a diverse range of biological activities, including anticancer activity. Studies have suggested that aloe emodin exhibited apoptosis in cancer cells and inhibited their proliferation [118,119]. However, aloe emodin itself is not sufficient for antitumor activity. It requires derivatization to synergize the potential of the final compound. Inspired by the pharmacological studies related to aloe emodin and various pharmacological properties of coumarin, Shang et al. designed and synthesized a series of aloe emodin–coumarin derivatives and tested their anticancer activity against various cancer cell lines. The results of the assay demonstrated that compound **40** was the most potent anticancer compound against the MCF-7 cancer cell lines, with an IC_50_ value of 1.56 µmol/L. The structure–activity relationship suggested that the compound containing aloe emodin with acetate is more potent against breast cancer cells than the aloe emodin parent structure [120]. Harmine, a representative of β-carboline alkaloids, is found in the seeds of the *Peganum harmala* plant and is known to possess a wide range of biological activities. Studies have demonstrated that harmine derivatives have different targets in anticancer treatment, such as the DNA, topoisomerase, kinases, and alpha-tubulin [121,122,123,124]. Pavić et al. synthesized a series of harmine–coumarin hybrids and evaluated their anticancer potential against various cancer cell lines. The results of the assay revealed that compound **41**, with an IC_50_ value of 1.9 µM against the MCF-7 cell line, was the most potent anti-breast cancer agent. The structure–activity relationship suggested that the substitution of the coumarin ring had the greatest influence on the activity of the compound. Fluorine substitution was beneficial for the activity, whereas the replacement of fluorine with hydrogen resulted in decreased activity [125]. A derivative inspired by ergosterol and coumarin (compound **42**), developed by Bu et al., showed efficacy against MCF-7 cells, with an IC_50_ value of 7.45 µM. An alkyl chain between coumarin and ergosterol was most suitable for the activity of the compound, while the replacement of the alkyl chain with a piperidine ring resulted in a decrease in the activity profile (Figure 15) [126].

### 3.12. Coumarin-Clubbed Benzothiazole/Benzodiazole Derivatives as Anti-Breast Cancer Agents

**Figure 16:**
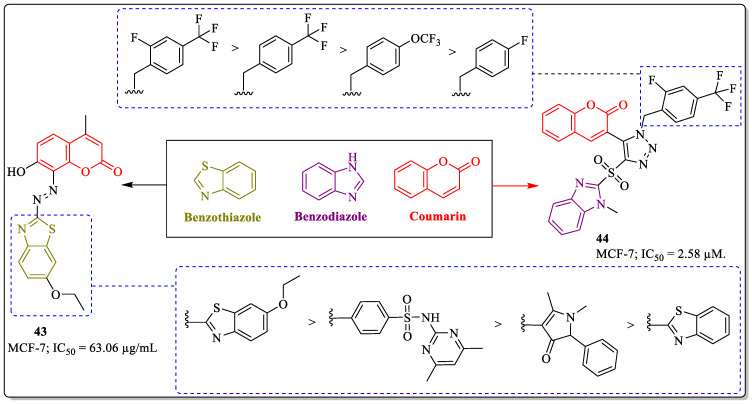
Coumarins clubbed with benzothiazole/benzodiazole with anti-breast cancer efficacy. MTT assay: compounds **43** and **44** were analysed after 48 h of incubation.

Benzothiazole belongs to the family of heterocycles and has the wide spectrum of biological activities [127]. Derivatives of benzothiazole were found to be good anticancer agents in various cancers, including breast cancer [128,129,130,131]. Inspired by the biological potential of benzothiazole and coumarin, Nagaraja et al. developed a series of 4-methylumbelliferone-containing heterocyclic compounds. The compounds were subjected to the evaluation of their anticancer profile. Among them, compound **43**, with an IC_50_ value of 63.06 µg/mL against the MCF-7, was found to be the most active anti-breast cancer compound. Compound **43** showed inhibitory potential against MMP-2 and 9, along with DNA cleavage activity on the pBR322 plasmid. The structure–activity relationship suggested that the substitution of 5-ethoxy-benzothiazole linked to coumarin via a hydrazine linkage was most suitable for the activity of the compound. The unsubstituted benzothiazole and the other substitutions resulted in a decreased cytotoxic potential of the structure [132]. Benzodiazole or Benzimidazole is the versatile scaffold used in the development of potent anticancer agents. Bendamustine, veliparib, and pracinostat are anticancer agents containing benzimidazole as the active pharmacophore. Inspired by triazole, benzimidazole, and coumarin, Kumar et al. synthesized a series of 1,2,3-triazole hybrids containing coumarin and sulfonyl-benzimidazole pharmacophores. Out of them, compound **44** was most effective against MCF-7 cells, with an IC_50_ value of 2.58 µM. The introduction of an electron-withdrawing group at the benzyl ring linked to triazole had a great influence on the activity of the compound. The presence of fluorine at the *ortho* position and the trifluoro group at the *para* position were also beneficial for the activity of the compound. The replacement of the substitutions with fluorine or oxy trifluoro led to decreased activity (Figure 16) [133].

### 3.13. Coumarin-Clubbed Diazole and Triazole Hybrids as Anti-Breast Cancer Agents

**Figure 17:**
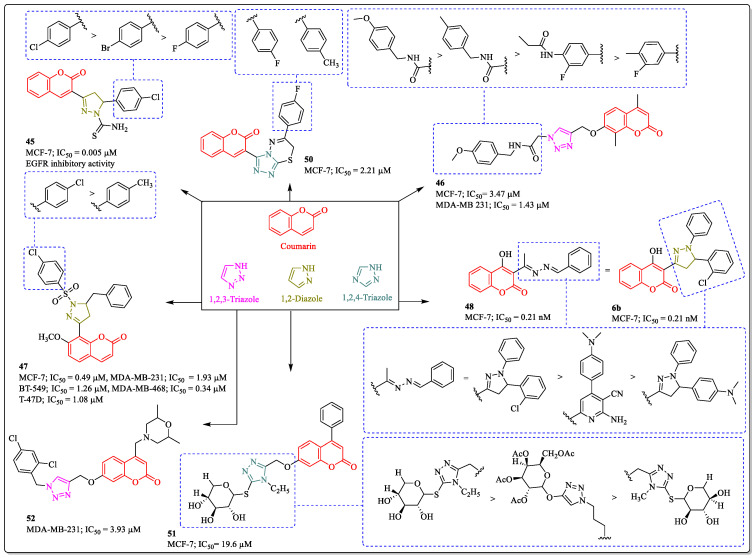
Coumarin-clubbed diazole, triazole hybrids with anti-breast cancer efficacy. MTT assay: compounds **46**–**50** were analysed after 24 h of incubation. Compounds **45**, **51**, and **52** were analysed after 48 h of incubation.

1,2,3-triazole, 1,2,4-triazole, and diazole belongs to the similar class of heterocycles. These basic scaffolds are widely used in the medicinal chemistry for drug development, and they are especially used in the molecular hybridization technique, in which two pharmacophores are connected via these heterocycles. Along with that, they also participate in the synergistic effect of the compound by establishing the interaction with the receptor amino acids. Ragab et al. designed and synthesized series of coumarin–pyrazoline hybrids and investigated their anticancer potential against various cancer cell lines. Among the whole series, compound **45** was effective against MCF-7 cells, with an IC_50_ value of 0.005 µM. The substitution of the chlorine group at the *para* position of the phenyl ring linked to diazole was beneficial for the activity of the compound, whereas the replacement of chlorine with bromine or fluorine led to decreased activity [134]. Similarly, a triatomic flexible agent bearing coumarin and triazole (compound **46**), developed by Ihmaid et al., showed efficacy against MCF-7 cells, with an IC_50_ value of 3.47 µM. The substitution pattern on the benzyl ring had the greatest influence on the activity of the compound. The substitution of the methoxy group at the *para* position of the benzyl ring was beneficial for the activity, whereas the replacement with the methyl group led to a decrease in the activity profile. The activity also decreases with the introduction of any substituent on the meta position of the benzyl ring [135]. A coumarin–pyrazoline hybrid (compound **47**), developed by Amin et al., showed anti-breast cancer activity, with IC_50_ values of 0.49 µM, 1.93 µM, 1.26 µM, 0.34 µM, and 1.08 µM against the MCF-7, MDA-MB-231, BT-549, MDA-MB-468, and T-47D cell lines. The substitution of chlorine at the *para* position of the phenyl ring linked to the sulphur atom was best suited for the activity of the compound, while the replacement of chlorine with the methyl group led to decreased activity [136]. A 3-Substituted-4-hydroxy coumarin derivative (compound **48**) developed by Latif et al. showed efficacy against MCF-7 cells, with an IC_50_ value of 0.21 nM [137]. A coumarin–triazolothiadiazine conjugate (compound **50**), developed by Iqbal et al., showed efficacy against MCF-7 cells, with an IC_50_ value of 2.21µM. The substitution of fluorine at the *para* position of the phenyl ring was best suited for the activity of the compound, while the replacement of fluorine with the methyl group significantly decreased activity [138]. A triazole–coumarin–glycoside hybrid (compound **51**), developed by the El-Sayed et al., showed effectiveness against MCF-7 cells, with an IC_50_ value of 19.6 µM. The substitution of 1,2,4-triazole-thioglycosides at the coumarin linked by an ether bridge was beneficial for the activity of the compound. The replacement of 1,2,4-triazole with the 1,2,3-triazole ring results in a decrease in the activity of the compound. On the other hand, the replacement of the ethyl group on the triazole ring with the methyl ring results in decreased activity [139]. Similarly, a morpholine-linked coumarin–triazole hybrid (compound **52**) developed by Goud et al. showed efficacy against hormone negative MDA-MD-231, with an IC_50_ value of 3.93 µM (Figure 17) [140].

### 3.14. Coumarin-Clubbed Thiazole Derivatives as Anti-Breast Cancer Agents

**Figure 18:**
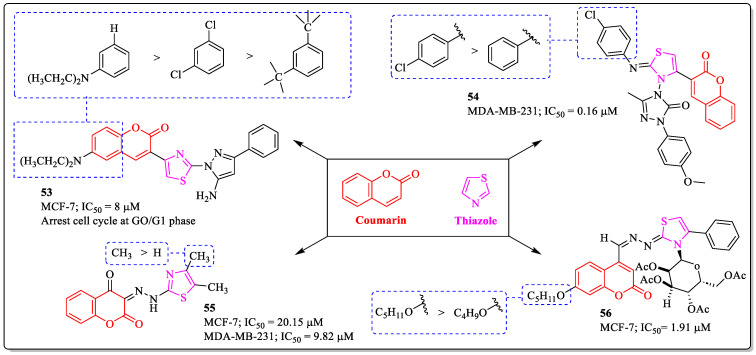
Coumarin-clubbed thiazole hybrids with anti-breast cancer efficacy. MTT assay: compounds **53**–**56** were analysed after 72 h of incubation.

Thiazole is the privileged scaffold in medicinal chemistry. Its moiety is present in a large number of natural products, as well as in marketed drugs. Recent studies showed that nitrogen-rich compounds displayed a good chemotherapeutic action [141]. Vaarla et al. designed and synthesized series of 3-(2-5-amino-3-aryl-1*H*-pyrazol-1-yl)thiazol-4-yl)-2*H*-chromene-2-ones, and compound **53** among them was the most potent, with an IC_50_ value of 8 µM against MCF-7 breast cancer cells. The substitution of *N*,*N*-dimethyl group at the sixth position of the coumarin ring was beneficial for the activity of the compound, while with the replacement of the *N*,*N*-dimethyl group with the chlorine atom, the activity decreased [142]. Similarly, Shaikh et al. synthesized a series of coumarin-3-yl-thiazol-3-yl-1,2,4-triazolin-3-ones and evaluated them for their anticancer activity. Among them, compound **54** was found to be the most potent anti-breast cancer agent, with an IC_50_ value of 0.16 µM against MDA-MB-231 breast cancer cells. The structure–activity pattern revealed that the *para* chlorine-substituted phenyl ring linked to the thiazole was best suited for the activity of the compound. The unsubstituted phenyl ring leads to decreased activity [143]. A series of isooxazole and thiozolohydrazinylidene chromen-2,4-diones were developed by Jashari et al. and evaluated against breast cancer cells. Compound **55** was most effective, with an IC_50_ value of 9.82 µM against MDA-MB-231 cells. The structure–activity relationship suggested that the presence of a methyl group on the thiazole ring was essential for the activity; with the removal of the group with the hydrogen atom, the activity decreases [144]. Toan et al. designed and synthesized a series of thiazoline–coumarin hybrids and evaluated them for their anticancer activity. The results of the assay demonstrated that compound **56**, with an IC_50_ value of 1.91 µM against the MCF-7 cell line, was the most potent compound in the series. The structure–activity relationship revealed that the substitution of the alkyl chain up to five carbons on the coumarin via an ether linkage was beneficial for the activity of the compound, whereas with the decrease in the length of the chain, the activity decreased (Figure 18) [145].

### 3.15. Coumarin-Clubbed Oxadiazole Derivatives as Anti-Breast Cancer Agents

**Figure 19:**
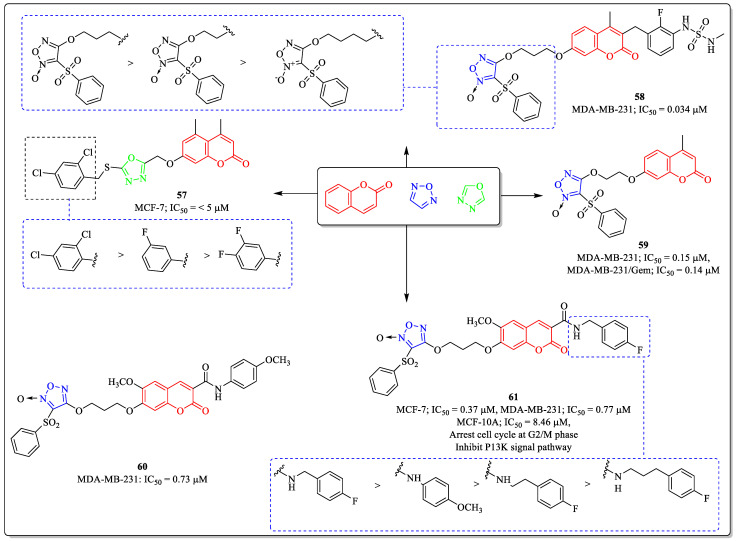
Coumarin-clubbed oxadiazole hybrids with anti-breast cancer efficacy. MTT assay: compounds **57**–**61** were analysed after 48 h of incubation.

1,3,4-oxadiazole and 1,2,5-oxadiazole are the isomers of oxadiazole and are extensively used in medicinal chemistry drug development due to their diverse biological potential. The scaffold is responsible for the lipophilicity of the final molecule. Derivatives containing oxadiazole moiety are known to show anticancer activity. Inspired by the potential of the oxadiazole and coumarin, Dhawan et al. designed and synthesized coumarin-tagged 1,3,4-oxadiazole conjugates, and their anticancer potential was evaluated. Among them, compound **57** was found to be effective toward MCF-7 cells, with an IC_50_ value of <5 µM. The substitution of the chlorine atoms at the *ortho* and *para* positions of the benzyl ring was best suited for the activity of the compound, whereas the replacement of chlorine with fluorine on the *meta* position or *meta* and *para* positions resulted in decreased activity [146]. A series of coumarin derivatives were synthesized by Carneiro et al. and evaluated for their anticancer potential. Most of the compounds displayed remarkable profiles. Among them, compound **58** was effective, with an IC_50_ value of 0.0034 µM against MCF-7 breast cancer cells. The length of the chain between coumarin and oxadiazole had a great influence on the activity of the compound. The length of the chain up to three carbons is optimum for the activity, with the increase or decrease in the carbon chain length leading to a decrease in activity [147]. Similarly, inspired by the pharmacological properties of coumarin and oxadiazole, Liu et al. designed and synthesized hybrids of phenylsulfonylfuronan and coumarin as potent anticancer agents and evaluated them for their anticancer activity against breast cancer cell lines. Among them, compound **59** was found to be the most potent anti-breast cancer agent, with IC_50_ values of 0.15 µM and 0.14 µM against MDA-MB-231 and MDA-MB-231/gem cell lines [148]. A coumarin derivative (**60**) developed by Yu et al. showed effectiveness against MDA-MB-231 cells, with an IC_50_ value of 0.73 µM, and, from the same series, compound **61** showed efficacy against MCF-7 cells, with an IC_50_ value of 0.37 µM. Compound **61** also arrests the cell cycle at the G2/M phase and inhibits the P13K signal pathway. The substitution of a fluorine atom at the *para* position of the phenyl ring in compound **61** was beneficial for the activity of the compound, while the replacement with the methoxy group led to a decrease in the activity profile. The addition or elimination of carbon chain length between the amide linkage and the phenyl ring also led to decreased activity (Figure 19) [149].

### 3.16. Fused Coumarin Derivatives as Anti-Breast Cancer Agents

**Figure 20:**
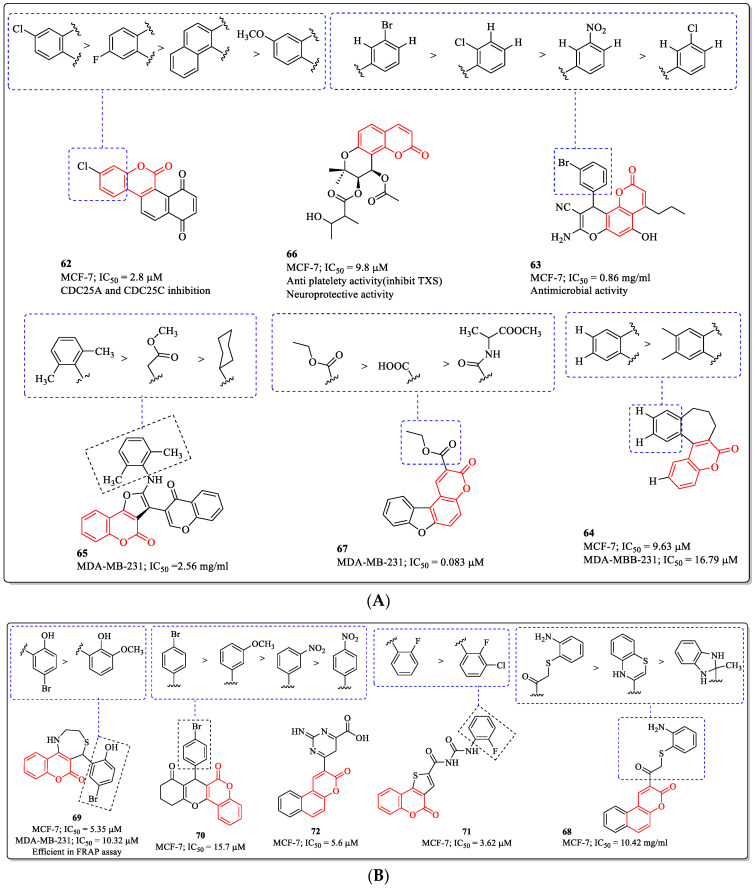
(**A**) Fused coumarin derivatives with anti-breast cancer efficacy. MTT assay: compound **63** was analysed after 24 h of incubation. Compounds **61**, **66**, and **67** were analysed after 48 h of incubation. Compound **62** was analysed after 72 h of incubation. (**B**) Fused coumarin derivatives with anti-breast cancer efficacy. MTT assay: compounds **69** and **72** were analysed after 24 h of incubation. Compounds **70** and **71** were analysed after 72 h of incubation.

Fused coumarins have attracted the scientific community due to their diverse range of pharmacological properties. This literature survey highlightes that coumarin-fused derivatives, including coumarin-fused coumarins, bis-coumarins, and fused coumarin, are important classes in drug development. These derivatives have shown outstanding potential in interdisciplinary areas. Zwergel et al. designed and synthesized coumarin and quinolinone-based polycycles and investigated their anticancer activity. Compound **62** from the series was found to be most effective, with an IC_50_ value of 2.8 µM against MCF-7 cells. The substitution of chlorine at the sixth position of the coumarin ring is desirable for the activity of the compound. The replacement of chlorine with fluorine, methoxy, or unsubstituted coumarin resulted in decreased activity [150]. Similarly, a 5,7-dihydroxy-4-propyl-2*H*-chromen-2-one derivative (compound **63**) developed by Abd-El-Aziz et al. showed efficacy against MCF-7 cells, with an IC_50_ value of 0.86 µg/mL. The substitution of bromine at the *para* position of the phenyl ring was best suited for the activity of the compound, while the replacement of the bromine group with the nitro or chlorine substituents resulted in decreased activity [151]. A series of benzosuberone-bearing coumarins were developed by Yadagiri et al. and evaluated for their anticancer activity against various cell lines. Among them, compound **64** was found to be most potent anti-breast cancer agent, with IC_50_ values of 9.63 µM and 16.79 µM against the MCF-7 and MDA-MB-231 breast cancer cell lines. The structure–activity relationship suggested that no substitution on benzosuberone was beneficial for the breast cancer activity, while substituting methyl groups on benzosuberone significantly decreased the activity of the compound [152]. Meydani et al. synthesized series of furopyranone and furocoumarin chromone conjugates, and their anticancer activity was evaluated on the breast cancer cells. Among them, compound **65** was most effective toward MDA-MB-231 cells, with an IC_50_ value of 2.56 µg/mL [153]. Similarly, another coumarin derivative (compound **66**), developed by Mira et al., showed efficacy against MCF-7 cells, with an IC_50_ value of 9.8 µM [154]. A benzofuran–coumarin analogue (**67**) developed by Francisco et al. showed efficacy against MDA-MB-231 cells, with an IC_50_ value of 0.083 µM. The substitution of the methyl acetate group on the coumarin ring was best suited for the activity of the compound, while the replacement of methyl acetate with the carboxylic acid group led to decreased activity [155]. Mohamed et al. designed and synthesized Benzo[f]coumarin derivatives as anticancer agents. The results of the test revealed that compound **68**, with an IC_50_ value of 10.42 µg/mL against the MCF-7 cell line, was the most potent anti-breast cancer agent. The structure–activity relationship suggested that the substitution of the ortho amino phenyl ring via sulphur linkage was advantageous for the activity of the compound, while the replacement of the phenyl ring with the thiazine ring resulted in diminished activity [156]. A 1,4-Thiazepine derivative (**69**) developed by Khoobi et al. showed efficacy against MCF-7 and MDA-MB-231 cells, with IC_50_ values of 5.35 µM and 10.32 µM. The substitution of bromine at the fifth position and the hydroxy group at the second position of the phenyl ring were desirable for the activity of the compound, whereas the introduction of a methoxy group at the third position and the removal of the bromine atom led to a decrease in its activity [157]. Similarly, a benzopyrano[3,2-c]chromene-6,8-dione derivative (**70**) developed by Shafiee et al. showed efficacy against MCF-7 cells with, an IC_50_ value of 15.7 µM. The substitution of the bromine atom at the *para* position of the phenyl ring was desirable for activity, while the replacement of bromine with the other substituent led to a decrease in activity [158]. A coumarin [3,2-c]thiophene derivative (**71**) developed by Wittine et al. showed efficacy against MCF-7 cells, with an IC_50_ value of 3.62 µM. The substitution of fluorine at the *ortho* position of the phenyl ring was best suited for the activity of the compound, while the introduction of chlorine at the *meta* position led to a decrease in its activity [159]. Inspired by the pharmacological properties of coumarin, Salem et al. synthesized a series of coumarin derivatives and evaluated their anti-breast cancer activity. The results of the assay suggested that compound **72**, with an IC_50_ value of 5.6 µM against MCF-7 cell line, was the most potent candidate (Figure 20A,B) [160].

### 3.17. Coumarin-Appended Selenium Derivatives as Anti-Breast Cancer Agents

**Figure 21:**
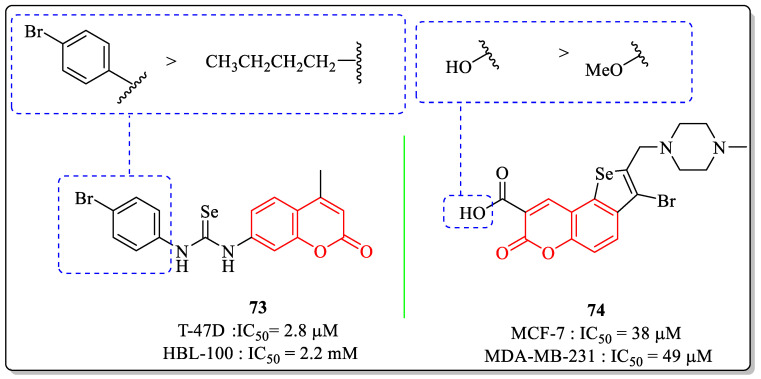
Coumarin-appended selenium derivatives with anti-breast cancer efficacy. MTT assay: compound **73** was analysed after 24 h of incubation. Compound **74** was analysed after 72 h of incubation.

Lagunes et al. designed and synthesized a series of selenium-containing coumarin derivatives and investigated their anticancer potential. Among all the synthesized derivatives, compound **73**, with IC_50_ values of 2.2 µM and 2.8 µM against the HBL-100 and T-47D breast cancer cell lines, was the most potent agent. The substitution of bromine at the *para* position of the phenyl ring was best suited for the activity, whereas the replacement of the phenyl ring with the alkyl chain resulted in decreased efficacy [161]. A seleno[2,3-f]coumarin derivative developed by Arsenyan et al. showed anti-breast cancer efficacy, with IC_50_ values of 38 µM and 49 µM against the MCF-7 and MDA-MB-231 cell lines. The substitution of the carboxylic acid group at the third position of the coumarin ring was desirable for the activity, while the replacement of the hydroxyl group of carboxylic acid with the methoxy group led to decreased activity (Figure 21) [162].

### 3.18. Coumarin-Appended Sulphoxide Derivatives as Anti-Breast Cancer Agents

**Figure 22:**
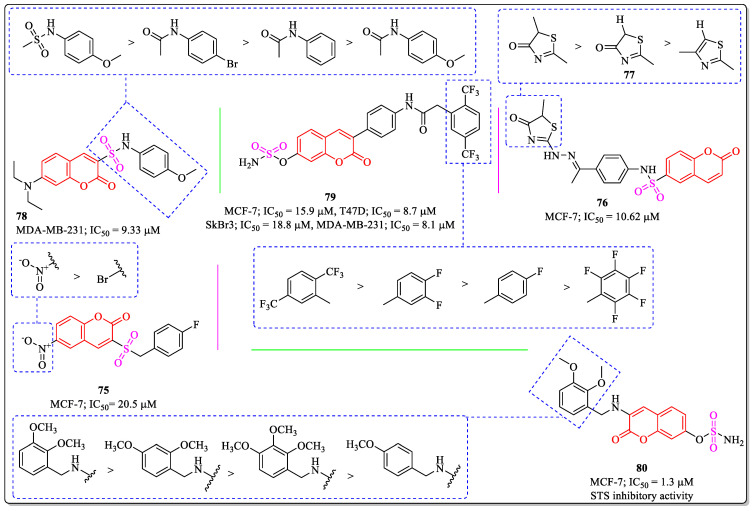
Coumarin-appended sulphoxide derivatives with anti-breast cancer efficacy. MTT assay: compound **75** was analysed after 48 h of incubation. Compound **76** was analysed after 72 h of incubation. Compound **79** was analysed after 120 h of incubation. Sulforhodamine B (SRB) assay: compound **76** was analysed after 72 h of incubation.

A benzylsulfone coumarin derivative (**75**) developed by Wang et al. showed efficacy against MCF-7 cells, with an IC_50_ value of 20.5 µM. The substitution of the nitro group at the coumarin nucleus was beneficial for the activity of the compound, while the replacement of the nitro group with electronegative bromine led to decreased activity [163]. Sabt et al. designed and synthesized a series of coumarin-6-sulfonamides and evaluated their anticancer activity against various cancer cell lines. The results of the assay revealed that compound **76**, with an IC_50_ value of 10.62 µM against the MCF-7 cell line was the most potent anti-breast cancer agent. The substitution of a methyl group on the thiazole ring was desirable for the activity of the compound, while the replacement of the methyl group with hydrogen (**77**) diminished its activity. The presence of the carbonyl group on the thiazole was also essential for activity [164]. The coumarin–sulfonamide and coumarin–amide derivatives designed by Zhang et al. showed inhibitory potential against breast cancer cells. Among them, compound **78** was found to be most effective, with an IC_50_ value of 9.33 µM against the MCF-7 cell line. The 4-Methoxy phenyl sulfonamide group at the third position of coumarin was desirable for the activity of the compound, while the replacement of the sulphonamide group led to a decrease in its efficacy [165]. A coumarin derivative (**79**) developed by Dasko et al. also showed anti-breast cancer efficacy, with IC_50_ values of 15.9, 8.7, 18.8, and 8.1 µM against the MCF-7, T47D, SkBr3, and MDA-MB-231 cell lines [166]. Similarly, 3-benzylamino coumarin-7-O-sulfamate derivatives (compound **80**) developed by Hng et al. showed efficacy toward MCF-7 cells, with an IC_50_ value of 1.3 µM. The substitution of the methoxy group at the *ortho* and *meta* position of the benzyl ring adjacent to the amine linkage was desirable for the activity of the compound, while a change in the position or the removal of the methoxy group led to a decrease in its activity (Figure 22) [167].

### 3.19. Coumarin-Appended Phosphorus Derivatives as Anti-Breast Cancer Agents

**Figure 23:**
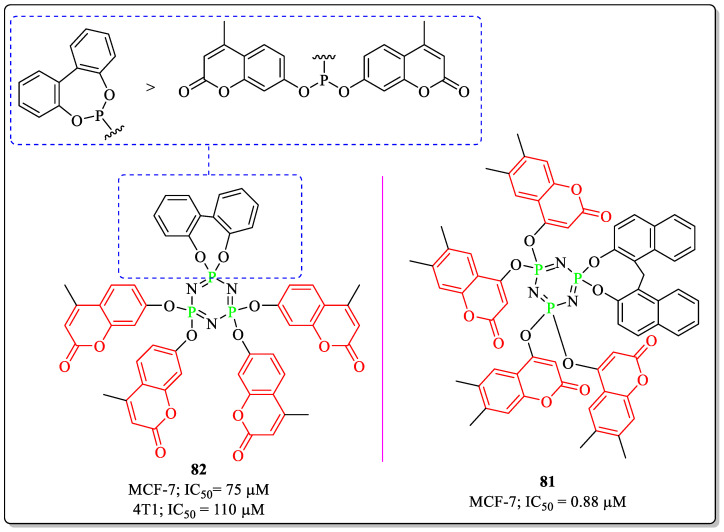
Coumarin-appended phosphorus derivatives with anti-breast cancer efficacy. MTT assay: compound **81** was analysed after 24 h of incubation. STS inhibition assay: compound **80** was analysed after 1 h of incubation.

Eker et al. designed and synthesized phosphorus-containing coumarin derivatives and evaluated them for their anticancer potential against various cancer cell lines. Among them, compound **81** was the most effective against MCF-7 cells, with an IC_50_ value of 0.88 µM [168]. Cyclo-triphosphazene functionalized with 4-methyl-7-hydrocoumarins (compound **82**), developed by Chen et al., showed efficacy toward MCF-7 cells, with an IC_50_ value of 75 µM. The substitution of the biphenyl rings linked to the phosphorus through an ether linkage was best suited for the activity of the compound, while the replacement of the biphenyl rings with the bis-coumarin moieties resulted in decreased activity (Figure 23) [169].

### 3.20. Miscellaneous Coumarin Derivatives as Anti-Breast Cancer Agents

**Figure 24:**
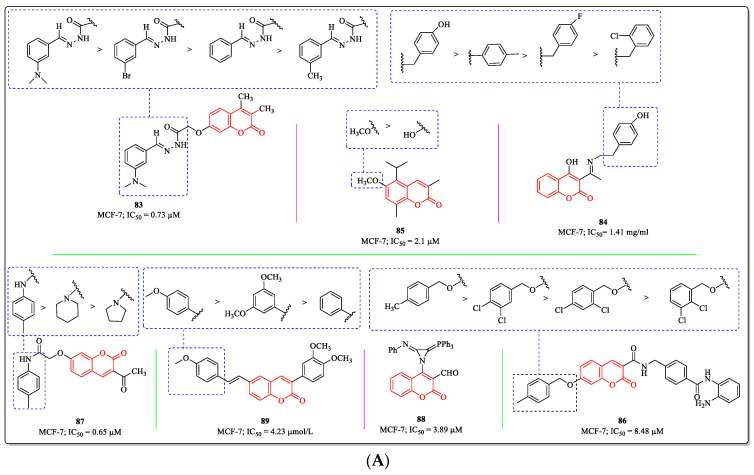

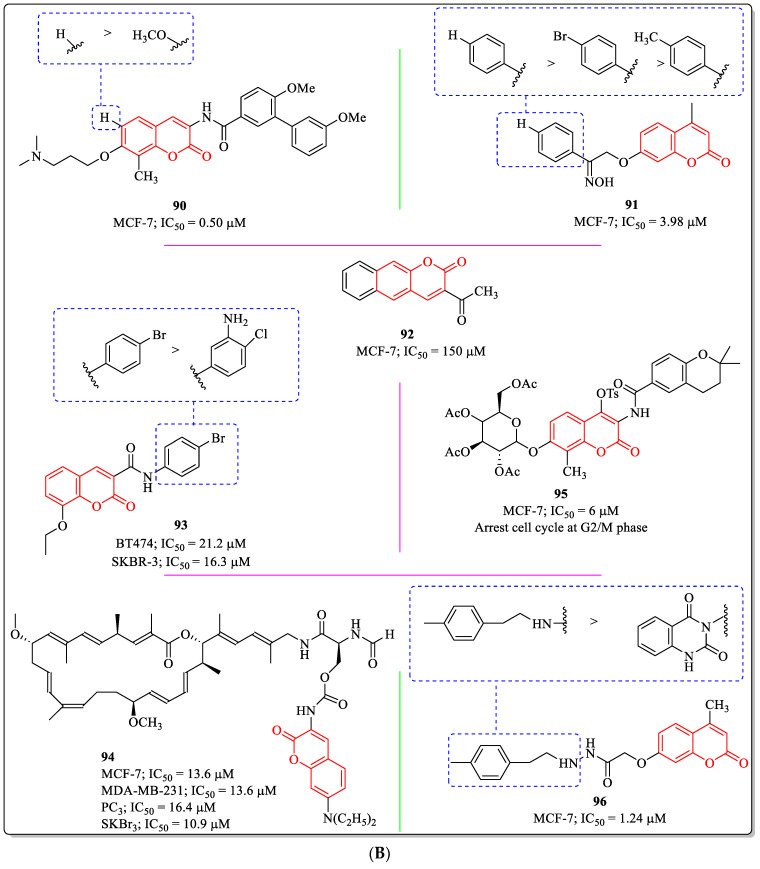

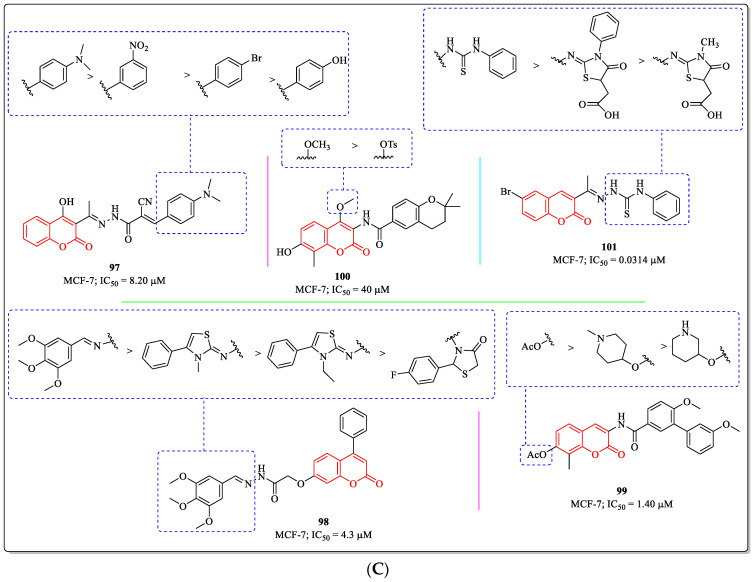
(**A**) Miscellaneous coumarin derivatives with anti-breast cancer efficacy. MTT assay: compounds **83**, **84**, and **86** were analysed after 48 h of incubation. Sulforhodamine B (SRB) assay: compound **89** was analysed after 48 h, and **85** and **88** were analysed after 72 h of incubation. (**B**) Miscellaneous coumarin derivatives with anti-breast cancer efficacy. MTT assay: compound **91** was analysed after 48 h, **95** after 48 h, and **94** and **95** after 72 h of incubation. Sulforhodamine B (SRB) assay: compound **89** was analysed after 48 h of incubation. (**C**): Miscellaneous coumarin derivatives with anti-breast cancer efficacy. MTT assay: compound **99** was analysed after 48 h of incubation. Compounds **97** and **98** were analysed after 48 h of incubation. Compound **100** was analysed after 72 h of incubation. Colorimetric method: compound **101** was analysed after 48 h of incubation.

Inspired by the pharmacological properties of coumarin and as an attempt to develop a promising anticancer therapy for inhibiting the VEGFR-2/AKT pathway, Ghany et al. designed and synthesized a series of coumarin-bearing aromatic hydrazone terminals at the seventh position. The synthesized compounds were evaluated for their anticancer potentials against various cell lines. The results of the assay suggested that compound **83** was the most potent candidate against the MCF-7 breast cancer cell line, with an IC_50_ value of 0.73 µM. The substitution of dimethylamine group at the *para* position of the phenyl ring was beneficial for the activity of the compound, while the replacement of dimethylamine with the bromine, hydrogen, or methyl group led to decrease in its activity profile [170]. A 4-Hydroxycoumarin-derived imine derivative (**84**) developed by Vaseghi et al. showed activity against MCF-7 cells, with an IC_50_ value of 1.41 µg/mL. The substitution of the hydroxyl group at the *para* position of the benzyl ring was best suited for the activity of the compound, while the replacement of the hydroxyl group with methyl or fluorine led to decreased activity. The activity also decreased with a change in the position of substitution [171]. Baghdadi et al. designed and synthesized series of coumarin and related o-nathoquinones. Among them, compound **85** was found to be the most potent against MCF-7 cells, with an IC_50_ value of 2.1 µM. The presence of the methoxy group at the fifth position was more beneficial than the hydroxyl group on the coumarin ring [172]. A coumarin-based benzamide derivative (compound **86**) developed by Abdizadeh et al. showed efficacy against MCF-7 cells, with an IC_50_ value of 8.48 µM. The substitution of the methyl group at the *para* position of the benzyl ring linked to the coumarin ring via an ether linkage was beneficial for the activity of the compound, while the replacement of methyl group with chlorine led to a decrease in its activity. The position of the substitution had a great influence on the potential of the compound, and the *para* position was the most favourable for its activity [173]. A 3,7-Disubstituted benzopyrone derivative (compound **87**) developed by Durgapal et al. showed effectiveness against MCF-7 cells, with an IC_50_ value of 0.65 µM. The substitution of the phenyl ring with the *para* methyl group, linked to the coumarin ring via an amide linkage, was best suited for the activity of the compound, while the replacement of the ring with the piperidine or pyrrolidine ring decreased its activity [174]. A chromenone derivative (compound **88**) developed by Abd-El-Maksoud et al. showed desirable efficacy toward MCF-7 cells with, an IC_50_ value of 3.89 µM [175]. Similarly, a resveraterol–coumarin hybrid (**89**) developed by Amin et al. showed efficacy against MCF-7 cells, with an IC_50_ value of 4.23 µM. The substitution of the methoxy group on the *para* position of the phenyl ring was desirable for the activity of the compound, while the substitution of the methoxy group at both the *meta* positions or lack of substitution led to a decrease in its potential [136]. A novobiocin analogue (compound **90**) developed by Zhao et al. showed efficacy against MCF-7 cells, with an IC_50_ value of 0.50 µM. No substitution at the sixth position and methyl substitution at the eighth position of the coumarin ring was beneficial for the activity against the MCF-7 cells, while the potency of the compound decreased with the substitution of the methoxy group at the sixth and eighth position of coumarin [176]. A sugar 4-methyl coumarin derivative (compound **91**) developed by Mohammed et al. showed efficacy against MCF-7 cells, with an IC_50_ value of 3.98 µM. An unsubstituted phenyl ring was found to be desirable for the activity of the compound, while the addition of electronegative substituents decreased the anticancer potential of the structure [177]. Inspired by the pharmacological properties of coumarin, Shylaja et al. designed and synthesized series of 3-acetyl-2*H*-benzo[g]chromen-2-one and evaluated their anticancer potential against the breast cancer cells. The results of the assay displayed the compound **92** as the most potent anti-breast cancer agent, an IC_50_ value of 150 µM against the MCF-7 cell line [178]. Taking lead from the promising biological profile of coumarin, Reddy et al. designed and synthesized series of coumarin-3-(*N*-aryl)carboxamides, which were investigated for their anticancer activity against breast cancer cell lines. Among them, compound **93** was found to be the most potent anti-breast cancer compound, with IC_50_ values of 21.23 µM and 16.3 µM against the BT474 and SKBR3 cell lines. The substitution of the bromine atom at the *para* position of the phenyl ring was essential for the activity of the compound, while the replacement of bromine with chlorine and the introduction of amine at the *meta* position led to a decrease in the activity of the resultant compound [179]. A carbamate derivative of iejimalide (compound **94**) developed by Schweitzer et al. showed anti-breast cancer efficacy, with the IC_50_ values of 13.6, 13.6, 16.4, and 10.9 µM against MCF-7, MDA-MB-231, PC3, and SKBR3 breast cancer cell lines [180]. Similarly, a novobiocin analogue (compound **95**) developed by Audisio et al. showed efficacy toward MCF-7 cells, with an IC_50_ value of 6 µM and arrested the cell cycle at the G2/M phase [181]. Another coumarin derivative (compound **96**) developed by Ahmed et al. exhibited efficacy toward MCF-7 cells, with an IC_50_ value of 1.24 µM. A benzyl ring linked by the hydrazine linkage was best suited for the activity, whereas the replacement of the benzyl ring with the uracil ring in the structure resulted in decreased activity [182]. Based on the previous studies of the potential of 4-hydroxycoumarinhydrazide derivatives, Pilli et al. designed and synthesized different acroloylcyano–hydrazone derivatives and investigated their anticancer activity. Among them, compound **97** was found to be most active toward MCF-7 cells, with an IC_50_ value of 8.20 µM. The substitution of the dimethyl amino group at the *para* position of the benzyl ring was good for the activity profile, while the replacement of the dimethyl amino group with the bromine or hydroxyl led to decreased activity. The activity of the compound also decreased with the change of substitution position; the meta position of the benzyl ring was not favourable for substitution [183]. Batran et al. designed and synthesized 4-phenyl coumarin derivatives and their anti-breast cancer activity was evaluated. Among all the synthesized compounds, **98** was the most effective toward MCF-7 cells, with an IC_50_ value of 4.3 µM. The substitution of the benzyl ring with the *meta*, *para* methoxy group was beneficial for the activity of the compound [184]. A sugar analogue of novobiocin (compound **99**), developed by Donnelly et al., showed efficacy toward MCF-7 cells, with an IC_50_ value of 1.40 µM. The substitution of the acetoxy group at the seventh position of coumarin was beneficial for the activity of the compound, while the replacement of the acetoxy group with the piperidine ring led to decreased activity [185]. Similarly, another novobiocin analogue (**100**) developed by Le et al. showed efficacy toward MCF-7 cells, with an IC_50_ value of 40 µM. The substitution of the methoxy group at the fourth position of coumarin was responsible for the desirable activity, while the activity decreased with the replacement of the methoxy group with the tosyl group [186]. A coumarin derivative (compound **101**) developed by Sarhan et al. showed efficacy toward the MCF-7 cells, with an IC_50_ value of 0.0314 µM. The ethyl hydrazone linker was found to be the most desirable for the activity of the compound, while the potency of the compound decreased with the introduction of a thiazole ring (Figure 24A–C) [187].

## 4. Conclusions

The emerging resistance among the available anti-breast cancer agents is forcing medicinal chemists to discover alternatives to the available drugs in clinical practice. Naturally occurring coumarins with anti-breast cancer efficacy not only provide new leads but also a platform to develop novel, potent, and safer anti-breast cancer agents. Researchers are continuously developing novel synthetic coumarin derivatives by taking leads from naturally occurring coumarins to target breast cancer and simultaneously explore novel coumarins and their potential against breast cancer. The data generated in this review elucidated the design strategies, mechanisms, and structure–activity relationships of 101 exclusive coumarin derivatives with potential anti-breast cancer efficacy. Careful analysis suggests some important pharmacophores and fragments which, in combination with the coumarin nucleus yield highly potent architectures to target breast cancer. Overall analysis suggests that the coumarin pharmacophore, in combination with piperazine (**1**), piperidine (**4**–**8**), chalcone (**23**), alkyl chain (**28**, **30**, **31**), pyrimidine (**32**, **33**), thiazole (**54**), and oxadiazole (**58**, **59**, **61**), has generated optimum derivatives with sub-micromolar IC_50_ values. These derivatives can thus be utilized for further refinement and the generation of new architectures to target breast cancer. These data can act as a skeleton for medicinal chemists in designing a new class of potent and safer coumarin derivatives for efficiently targeting breast cancer.
